# A universal chimeric antigen receptor (CAR)–fragment antibody binder (FAB) split system for cancer immunotherapy

**DOI:** 10.1126/sciadv.adv4937

**Published:** 2025-07-04

**Authors:** Ainhoa Arina, Edwin Arauz, Elham Masoumi, Karolina W. Warzecha, Annika Sääf, Łukasz Widło, Tomasz Slezak, Aleksandra Zieminska, Karolina Dudek, Zachary P. Schaefer, Maria Lecka, Svitlana Usatyuk, Ralph R. Weichselbaum, Anthony A. Kossiakoff

**Affiliations:** ^1^ Department of Radiation and Cellular Oncology and Ludwig Center for Metastasis Research, The University of Chicago, Chicago, IL, USA.; ^2^ Department of Biochemistry and Molecular Biology, University of Chicago, Chicago, IL, USA.; ^3^ Jagiellonian University, Kraków, Poland.; ^4^ Warsaw University of Technology, Warsaw, Poland.; ^5^ University of Warsaw, Warsaw, Poland.; ^6^ Silesian University of Technology, Gliwice, Poland.; ^7^ Wroclaw University of Science and Technology, Wrocław, Poland.; ^8^ Institute for Biophysical Dynamics, The University of Chicago, Chicago, IL, USA.

## Abstract

Chimeric antigen receptor (CAR) T cell therapy has shown extraordinary results in treating hematological cancer but faces challenges like antigen loss, toxicity, and complex manufacturing. Universal and modular CAR constructs offer improved flexibility, safety, and cost-effectiveness over conventional CAR constructs. We present a CAR–fragment antibody binder (Fab) platform on the basis of an engineered protein G variant (GA1) and Fab scaffolds. Expression of GA1CAR on human CD8^+^ T cells leads to antigen recognition and T cell effector function that can be modulated according to the affinity of the CAR for the Fab and of the Fab for the target. GA1CAR T cells can recognize multiple Fab-antigen pairs on breast and ovarian cancer cell lines. Adoptively transferred GA1CAR T cells control tumors in breast cancer xenograft models, and their targeting can be quickly redirected using different Fabs. This versatile “plug-and-play” CAR T platform has potential for application in personalized therapy, preventing antigen loss variant escape, decreasing toxicity, and increasing access.

## Teaser

A CAR T platform using engineered protein G variants and Fab scaffolds enables safe, adaptable, multi-targeted cancer therapy.

## INTRODUCTION

Chimeric antigen receptor (CAR) T cell therapy has demonstrated impressive success in the treatment of hematological malignancies, such as acute lymphoblastic leukemia and non-Hodgkin’s lymphoma, by genetically engineering T lymphocytes to target specific cancer antigens. Despite these advances, CAR T cell therapy faces several substantial challenges, including low tumor penetration, severe toxicities like tumor lysis syndrome, cytokine release syndrome, and CAR T–related encephalopathy syndrome (*1*–*3*). Many patients experience relapse due to antigen heterogeneity or loss (*4*–*10*), and the therapy’s application to solid tumors has been limited by factors such as low infiltration into the tumor microenvironment and high antigenic variability (*6*, *11*–*16*). Many adverse side effects stem from potent off target effects that induce killing of normal tissue that expresses a threshold amount of the target protein (*17*, *18*). Current CAR T treatments are highly personalized and complex, leading to high costs and limited accessibility. To address these issues, innovative strategies are needed to enhance safety, efficacy, and accessibility.

Effective CAR T cell therapy depends on manifold antigen engagement properties like antigen density, intermembrane distance, epitope location and orientation, and targeting moiety [single-chain variable fragment (scFv)], as well as its spacer length (*19*, *20*). Considering the number of interrelated parts, it is clear that no standard template can be followed to construct the optimal CAR T candidate. Factoring the timing considerations and cost of production, it is virtually impossible to undertake patient specific optimization of traditional CAR T constructs. With these considerations in mind, numerous efforts have been made to develop “universal” CAR T constructs based on modular, split, and switchable systems that allow precise control of the cell therapy’s timing, strength, and specificity (*21*). These systems cover a range of quite different approaches (*21*–*26*). However, these approaches still often involve complex designs with structural constraints.

To address this issue, described here is the development and testing of GA1CAR, a novel modular antigen receptor system that uses pairing of an engineered protein G variant (GA1) with a set of antibody fragment Fabs with different modifications in their scaffolds. In this system, the Fab module has dual functions: antigen recognition through the CDR loops of its variable domain and attachment to GA1 of the CAR through its constant domain. The affinities of either contact interface can be adjusted. By using modified Fab scaffolds having varying affinities to GA1, the GA1CAR can fine-tune therapeutic responses such as cytokine release, providing a mechanism to adjust the intensity of the immune response. Because GA1CAR T cells and Fab are delivered separately and bind in vivo, the interchangeable Fab scaffolds also provide an on-off switching capability and provide for the ability to assess the patient’s tolerance to the GA1CAR in a systematic way. A further attribute of the system is that the circulating GA1CAR T cells can be efficiently reloaded by injecting a Fab^LRT^ with the desired targeting capability. This allows for targeting multiple antigens by simply swapping out the particular Fab, offering a “plug-and-play” approach that enhances versatility in targeting different tumor antigens (*27*, *28*). Here, using xenograft mouse models, we determined the in vivo efficacy of GA1CAR T cells and the ability to redirect GA1CAR T cells from one target to another by treating the animals with Fabs targeting different tumor-specific antigens. Comparison of the phenotype of GA1 versus conventional scFv CAR T cells showed a higher activation and effector function upon contact with cancer cells for the GA1CAR T cells. Additionally, we show that the unloaded GA1CAR T cells can remain dormant in the animal for an extended period and then, in the case of tumor rebound, be reloaded by reinfusing the target appropriate Fab. Together, this novel modular CAR system, the GA1CAR, is endowed with higher versatility, flexibility, and safety advantages compared to standard scFv CARs and lower complexity than other modular CARs. It has demonstrated specificity for various antigens overexpressed in breast and ovarian cancers and shows promise in preclinical models for personalized therapy applications.

## RESULTS

### Design and generation of GA1CAR

**Fig. 1.**
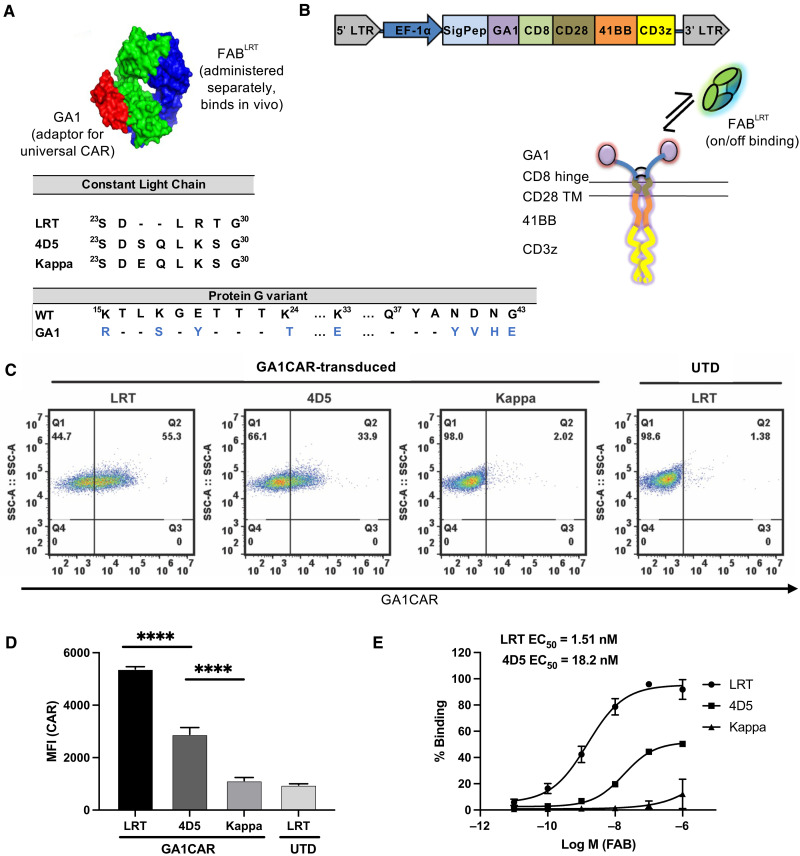
Design of the GA1CAR. (**A**) Cartoon showing binding of protein GA1 (red) and Fab^LRT^ scaffold (green for Lc and blue for Hc), as well as engineered mutations present on different Fab scaffolds and protein GA1. The GA1 mutations, shown in blue, increase binding to Fab and eliminate binding to endogenous IgG (*28*, *29*). (**B**) Schematic of lentiviral vector for GA1CAR expression and cartoon showing GA1CAR expressed on the cell surface and its Fab-capturing mechanism. TM, transmembrane. (**C**) Representative plots showing surface expression of GA1CAR in Jurkat cells (GA1CAR J) after lentiviral transduction, determined by flow cytometry after incubation with an irrelevant Fab^LRT^ (anti-MBP) followed by a secondary antihuman F(ab′)_2_ antibody conjugated to Alexa Fluor 647. Fab^LRT^ were used at 200 nM. UTD, untransduced. (**D**) Quantification of binding of different Fab scaffolds to GA1CAR expressed on Jurkat cells by flow cytometry. MFI, mean fluorescence intensity. (**E**) EC_50_ determination of different Fab scaffolds binding to GA1CAR J. The data are presented as the means ± SD of four independent experiments (*n* = 4). Statistical significance was determined by Tukey’s multiple comparisons test after one-way analysis of variance (ANOVA) (****P < 0.0001).

The GA1CAR system is a modular chimeric antigen receptor that uses components coevolved by phage display technologies to function entirely orthogonal to all naturally occurring immunoglobulins (*27*, *28*). Protein G inherently binds to immunoglobulins. The GA1 variant was engineered to prevent binding to both the immunoglobulin Fc domain, as well as to naturally occurring antibodies of any species or light-chain isoform (Fig. 1A) (*28*, *29*). The Fab scaffolds are based on the Herceptin Fab framework (4D5), which is widely used in clinical applications due to its stability and low immunogenicity (*29*). The 4D5 Fab framework was modified by phage display to create two unnatural scaffolds, LRT and SQRT, that bind with high specificity to the GA1 variant. The LRT and SQRT and the wild-type (WT) Kappa scaffolds were produced by introducing specific mutations into the Fab’s constant light-chain domain (*27*, *28*). The interaction affinities between these Fab scaffolds and GA1 were measured using surface plasmon resonance, revealing dissociation constants (*K*_*d*_) of 100 pM for LRT, 10 nM for SQRT, and 50 nM for 4D5. Fabs with WT human Kappa light chains do not bind to GA1 and serve as negative control (*27*).

The schematic of the GA1CAR lentivirus construct and its modular components to form a second generation CAR assembly are illustrated in Fig. 1B. The GA1 module was fused upstream of a CAR T construct containing a CD8 hinge, CD28 transmembrane domain, 41BB co-stimulatory domain, and CD3ζ domain (fig. S1). The production of a functional GA1CAR was confirmed through lentiviral transduction in Jurkat cells (GA1CAT J), using Fabs that bind the GA1 module, followed by staining with a fluorescent antihuman F(ab′)_2_ antibody (Fig. 1, C and D). The LRT scaffold showed superior binding to GA1 compared to the others (Fig. 1E), consistent with the observed dissociation constants. A single-molecule pulldown assay was used to verify the dimerization of the CD8 hinge in the GA1CAR structure (fig. S2). Using three distinct pulldown strategies: anti–soluble *N*-ethylmaleimide–sensitive factor attachment protein (SNAP) antibody (fig. S2A), biotinylated peptide antigen (fig. S2B), and anti–yellow fluorescent protein pulldown (fig. S2C), we counted immobilized molecules and their photobleaching steps. The resulting distribution, predominantly showing one and two photobleaching steps, supports a dimeric stoichiometry for the CAR (*30*, *31*). Together, these data confirm successful generation and expression of GA1CAR on human T cell surfaces.

### Functional characterization of GA1CAR

To assess how affinity affects functional response amplitude toward a surface expressed antigen, we used human embryonic kidney (HEK) cells expressing maltose-binding protein (MBP) and a conformationally sensitive anti-MBP Fab engineered with an LRT scaffold (Fab^LRT^). This Fab was generated by phage display to bind to the surface-expressed MBP in a manner that depended on the concentration of added maltose (fig. S3) (*32*). Thus, the GA1CAR/anti-MBP Fab system allowed for the evaluation of effector cell activation in response to variable binding free energies between the Fabs and the antigen (depending on maltose concentration) and of GA1CAR for Fab scaffolds [LRT (100 pM) versus 4D5 (80 nM)]. The functional capacity of GA1CAR J cells was first measured by interleukin-2 (IL-2) release and CD69 expression using MBP-coated wells (fig. S4, A and B). Activation levels were higher with soluble anti-MBP LRT Fab compared to the same Fab with a 4D5 scaffold (*P* < 0.05) and insignificant with the nonbinding Kappa Fab and untransduced cells. Using HEK cells expressing MBP as stimulators, IL-2 release was dose dependent on Fab^LRT^ and Fab^4D5^ concentrations (fig. S4, C and D). Because the Fabs have identical affinities to the MBP target, the 15-fold difference in stimulation of IL-2 secretion between these LRT and 4D5 Fabs is attributed to their differing affinities for the GA1 module. IL-2 release by GA1CAR J cells decreased with increasing maltose concentration (fig. S4, E and F), suggesting that lower binding affinity between the Fab and its target directly leads to decreased CAR T activation.

**Fig. 2.**
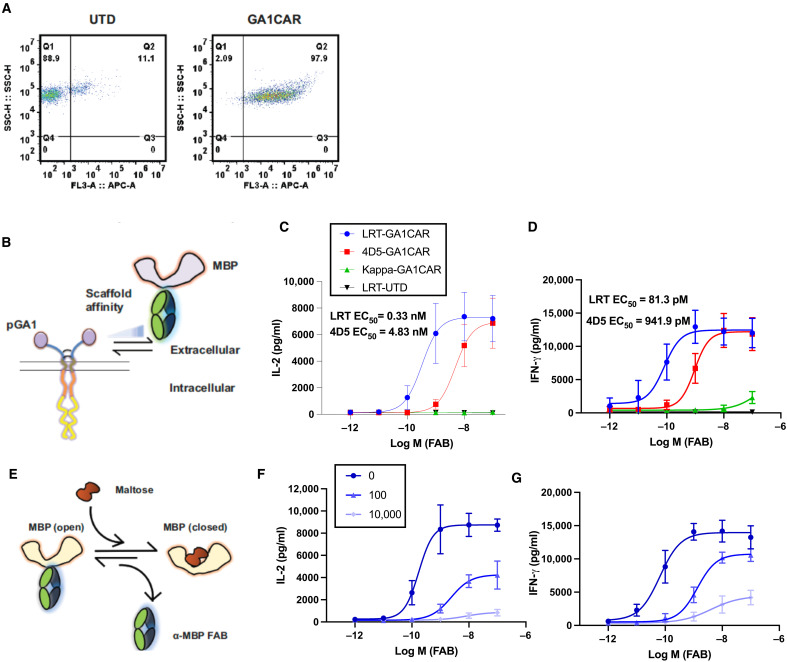
Tunable function of GA1CAR expressed in primary human CD8^+^ T cells. (**A**) Surface expression of GA1CAR in human CD8^+^ T cells, determined by flow cytometry after incubation with an irrelevant (anti-MBP) Fab^LRT^ followed by a secondary antihuman Fab antibody conjugated to Alexa Fluor 647. (**B**) Scheme for experiments determining the effect of using Fab scaffolds with varying affinities for the GA1CAR in GA1CAR T cell function: (**C**) IL-2 release and (**D**) IFN-γ release. The data are presented as the means ± SD; *n* = 3. (**E**) Scheme for experiments determining the effect of varying the affinity of the Fab for its target in GA1CAR T cell function, using the conformation-specific anti-MBP 70 Fab^LRT^ and HEK-MBP cells as targets. The affinity of the anti-MBP 70 Fab^LRT^ decreases with increasing maltose concentration. (**F**) IL-2 and (**G**) IFN-γ release. The data are presented as the means ± SD; *n* = 3.

The expression and functionality of GA1CAR were next evaluated in primary human CD8^+^ T cells. After lentiviral transduction, 40 to 98% of primary T cells achieved successful expression (Fig. 2A). In primary human CD8^+^ T cells transduced with GA1CAR (GA1CAR T), IL-2 release was significantly higher with high-affinity Fab^LRT^ [median effective concentration (EC_50_), 0.3 nM] as compared to lower-affinity Fab^4D5^ (EC_50_, 4.8 nM) when targeting HEK-MBP cells (Fig. 2, B and C). Similar results were observed for interferon-γ (IFN-γ) (Fig. 2D) and cytotoxicity (fig. S5) when comparing high- and low-affinity Fab scaffolds. Notably, based on the EC_50_ values of the Fab^LRT^ scaffold, IL-2 release was less sensitive than both IFN-γ (4-fold) and cytotoxicity (20-fold) as a functional readout. Maximum responses were achieved using Fab concentrations in range of the 0.1 to 10 nM. Using different maltose concentrations, we demonstrated a Fab-to-target affinity-dependent release of IL-2 and IFN-γ in GA1CAR T cells (Fig. 2, E to G). These data confirm the effective expression and regulatable function of GA1CAR on human primary CD8^+^ T cells and define the optimal range of Fab working conditions.

### Cytotoxicity of GA1CAR against various antigens and cancer cell lines

**Fig. 3.**
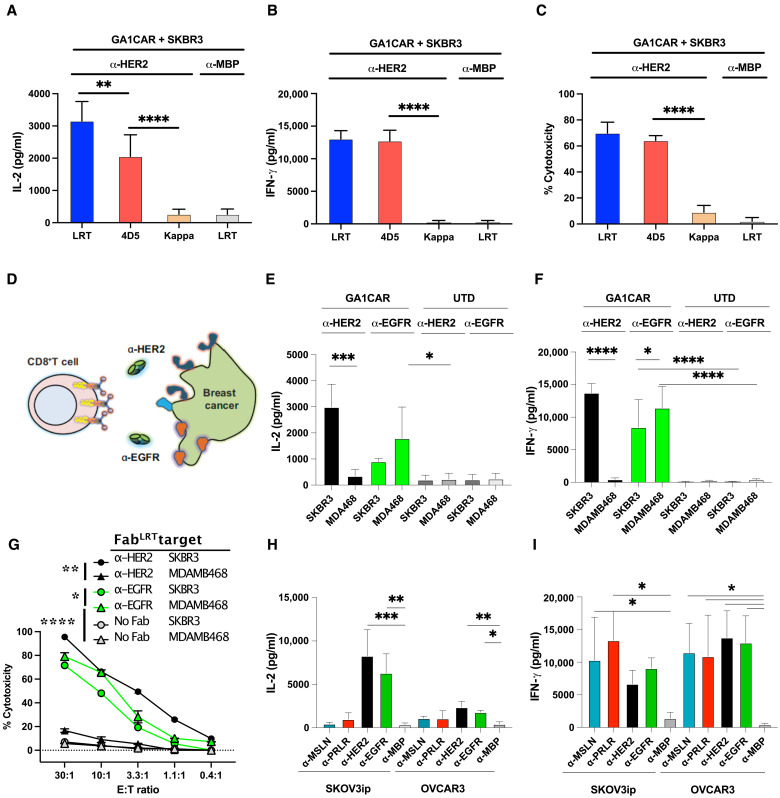
Recognition of multiple human cancer antigens by GA1CAR T cells. (**A** to **C**) GA1CAR T cell function using anti-HER2 Fab scaffolds with different affinities for GA1CAR and SKBR3 cells as targets: (A) IL-2 release, (B) IFN-γ release, and (C) cellular cytotoxicity. (**D**) Cartoon depicting the recognition of SKBR3 or MDA-MB-468 breast cancer cell lines by GA1CAR, using either anti-HER2 or anti-EGFR Fab^LRT^ (1 nM). (**E**) IL-2 release, (**F**) IFN-γ release, and (**G**) cellular cytotoxicity. (**H** and **I**) Recognition of SKOV3ip or OVCAR3 ovarian cancer cell lines by GA1CAR, using either anti-HER2, EGFR, PRLR, or MSLN Fab^LRT^ (1 nM). (H) IL-2 release and (I) IFN-γ release. Statistical significance was determined by one-way ANOVA followed by Šídák’s or Dunnett’s multiple comparisons tests for all panels except (G), where two-way ANOVA and Tukey’s multiple comparisons test were used. Statistical significance shown in (G) is for the most relevant comparisons at the 10:1 ratio. *P < 0.05; **P < 0.01; ***P < 0.001; ****P < 0.0001. Data shown as means ± SD; *n* = 3. Relative surface expression levels for the targets and cell lines used in this figure are shown in fig. S7. E:T ratio, effector-to-target ratio.

To evaluate the GA1CAR system in a cancer-associated antigen model, the SKBR3 cell line, known for high HER2 expression (fig. S6A), was used. Co-incubation of SKBR3 cells with GA1CAR T cells loaded with different anti-HER2 Fab scaffolds resulted in IL-2 and IFN-γ release and induced cytotoxicity when using the LRT or 4D5 scaffolds (*P* < 0.05), while only background levels were observed with the Kappa scaffold and anti-MBP-specific Fab (Fig. 3, A to C). Notably, scaffold affinity effects on T cell activation by SKBR3 cells were observed only for IL-2 release (LRT > 4D5 > Kappa) but not for IFN-γ or cytotoxicity, likely due to high antigen density masking these effects. Experiments with MDA-MB-231 cells with lower levels of HER2 expression than SKBR3 cells (fig. S6A) confirmed that IL-2 secretion was particularly sensitive to scaffold affinity (fig. S6B) and demonstrated that IFN-γ secretion (fig. S6C) could also be affected at lower levels of antigen stimulation, whereas cytotoxicity (fig. S6D) was unaffected. Further tests on breast cancer cell lines expressing epidermal growth factor receptor (EGFR) and HER2 at different levels demonstrated that GA1CAR T cells released IL-2 when engaged through anti-HER2 Fab^LRT^ and cocultured with SKBR3 cells (*P* < 0.05), but not with MDA-MB-468 cells (Fig. 3E) that lack HER2 (fig. S7A). Anti-EGFR Fab^LRT^ induced IL-2 release in both SKBR3 and MDA-MB-468 cell lines. Similar trends were observed for IFN-γ release and cytotoxicity (Fig. 3, F and G). Low EGFR expression on SKBR3 cells (fig. S7A) was sufficient for robust T cell activation (Fig. 3, F and G).

To explore additional cancer types, ovarian cancer cell lines SKOV3ip and OVCAR3 were used alongside Fabs targeting overexpressed receptors like MSLN, PRLR, HER2, and EGFR. GA1CAR T cells released IL-2 (Fig. 3H) and IFN-γ (Fig. 3I) in an antigen-specific way, even for antigens that showed very low staining levels by flow cytometry (MSLN and PRLR) (fig. S7B). These results highlight GA1CAR’s versatility across different antigen-Fab^LRT^ pairs in breast and ovarian cancer cell lines.

### On-off switching potential of interchangeable Fab scaffolds

A potential attribute of Fab scaffolds with varying affinities to the GA1 module is that they could be used to modulate GA1CAR activity through competitive displacement. To assess this potential, three Fab scaffolds with differing affinities—LRT (100 pM), SQLRT (10 nM), and 4D5 (50 nM)—were tested (*27*). The activation of GA1CAR T cells by SKBR3 cells through lower-affinity (SQLRT or 4D5) HER2 Fab scaffolds was quenched adding a non-targeting Fab with the higher-affinity LRT scaffold as a competitive blocker (fig. S8A). The effects on IFN-γ release and cytotoxicity were evaluated over time: 1, 2, 4, and 8 hours (fig. S8, B and C). Results indicate that the competitive Fab^LRT^ reduced cytotoxicity in a time- and affinity-dependent manner; SQLRT showed little quenching at later time points for IFN-γ release, suggesting rapid maximum activation post–CAR stimulation. These data demonstrate the potential to terminate GA1CAR T cell function using a non-targeting competitive Fab with a higher affinity scaffold.

### In vivo efficacy of GA1CAR in tumor xenograft mouse models

**Fig. 4.**
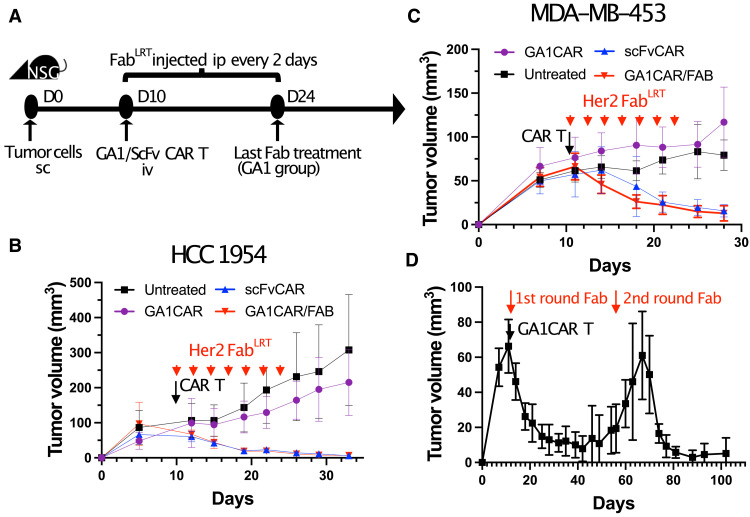
Antitumor activity of GA1CAR T cells in breast cancer xenograft models. (**A**) Experimental scheme. Female NSG mice were injected subcutaneously (sc) in the right flank with 5 × 10^6^ MDA-MB-453 or HCC1954 cells. Ten days later, the mice were injected with CAR T cells intravenously (iv) as indicated or left untreated. In the GA1CAR/FAB group, Fab^LRT^ was preincubated with the GA1CAR T cells for 30 min before GA1CAR T injection and then injected intraperitoneally (ip) every other day for 14 days at 200 μg per mouse. D, day. (**B** to **D**) Tumor growth curves of NSG mice bearing HCC1954 (B) or MDA-MB-453 [(C) and (D)] tumors. Data are representative from two independent experiments for each cell line, with five animals per group. Statistical significance was determined by two-way ANOVA followed by Dunnett’s multiple comparisons tests. The *P* values for GA1CAR/Fab and ScFv groups compared to GA1CAR control were *P* < 0.05 between days 19 to 33, *P* < 0.01 at day 26 for (B), and *P* < 0.05 between days 14 to 28, *P* < 0.01 between days 21 and 28 for (C).

Following the in vitro characterization of GA1CAR T cells, their antitumor activity was evaluated using an in vivo tumor xenograft mouse model. HER2-expressing HCC1954 cells (5 million) were injected subcutaneously into the right flank of immunocompromised NSG mice (Fig. 4, A and B). Once tumors were established, five mice per group were treated with GA1CAR T cells, either preincubated with HER2-specific Fab^LRT^ or not, and conventional scFv HER2-CAR T cells served as a control. Mice receiving GA1CAR T and Fab^LRT^ received additional Fab^LRT^ injections every 2 days for 2 weeks. Results showed that GA1CAR T cells combined with anti-HER2 Fab^LRT^ exhibited a robust antitumor effect (Fig. 4B). The activity of GA1CAR T cells with Fab^LRT^ was equivalent to that of conventional scFv CAR T against HER2 (*P* < 0.05 compared to GA1CAR control or untreated mice for both GA1CAR/Fab^LRT^ and ScFv groups), while tumors in control groups without CAR T cells or without Fab^LRT^ continued to grow.

### Multiple Fab dosing and tumor rebound

Although CAR T cell therapy can reduce tumor mass to undetectable levels, residual tumor cells can persist, leading to nucleation and regrowth. The potential to “reactivate” GA1CAR by reintroducing Fabs was tested to address the rebound phenomenon. MDA-MB-453 breast cancer cells were injected into NSG mice, using anti-HER2 Fab^LRT^ as a targeting agent (Fig. 4C). Initial injections began on day 10 and ended on day 24, resulting in rapid tumor contraction to baseline by day 27. Tumor volume was assessed every 3 days and remained stable until day 50, after which it increased sharply, indicating rebound (Fig. 4D). On day 60, five additional anti-HER2 Fab^LRT^ injections were administered, leading to a rapid reduction in tumor volume. These findings suggest that GA1CAR remains active and potent for at least 100 days and can be reloaded with Fab^LRT^ for extended immunological therapy.

### Pharmacokinetics of GA1CAR T cells

To characterize the in vivo dynamics of GA1CAR-Fab^LRT^ coupling, experiments were conducted comparing two delivery methods: preincubating anti-HER2 Fab^LRT^ with GA1CAR T cells before injection or delivering GA1CAR via retro-orbital injection followed by intraperitoneal HER2-Fab^LRT^ delivery. HCC1954 tumor volumes decreased similarly when comparing both delivery methods (fig. S9A), indicating that the GA1CAR and Fab^LRT^ engage and couple efficiently in circulation upon injection. The duration of GA1-Fab^LRT^ interaction was assessed by measuring Fab^+^ GA1CAR T cell percentages in peripheral blood post–Fab^LRT^ delivery. Eighteen hours after the first dose, comparable binding levels were observed regardless of preincubation (fig. S9B). However, 2 days after the third dose, no Fab^+^ GA1CAR T cells were detected (fig. S9B), although GA1CAR T cells persisted across all groups (fig. S9C). More detailed kinetic studies suggest that maximum Fab^LRT^ binding to GA1CAR in vivo occurs between 2 and 24 hours after a single intraperitoneal Fab^LRT^ injection and that Fab^LRT^ detaches from the GA1CAR by 60 hours after injection (fig. S9, D and E), although, in 2 of the 10 animals, the Fab^LRT^ could not be detected already at 24 hours (fig. S9, D and E). The interindividual variability in kinetics observed could be attributed at least, in part, to the use of the intraperitoneal route of administration for delivery of Fab^LRT^.

The properties of CAR T cells can be influenced by the peripheral blood mononuclear cell (PBMC) donors from which they are derived (*33*–*35*). To compare the effectiveness and kinetics of circulating GA1 versus scFv CAR T cells, CD8^+^ T cells from three donors were transduced with either GA1CAR or scFv CARs, and their impact on tumor growth and circulating levels was compared using three recipient mice per CAR T type and donor (**f**ig. S10A). Donor-matched GA1CAR T/HER2 Fab^LRT^ and scFv CAR T cells showed similar efficacy in controlling HCC1954 tumor growth, whereas tumors in mice treated only with GA1CAR T cells or Fab^LRT^ grew progressively (fig. S10B). Circulating CAR T levels were more affected by donor origin than CAR type and decreased around the time of tumor regression for two of the three donor cohorts (fig. S10C). CAR T cell levels from donor 1 increased after day 11 for both scFv and GA1CAR T recipients, leading to adverse effects requiring euthanasia due to a potential graft-versus-host effect (i.e., donor specific and not tumor related). Overall, GA1CAR T demonstrates similar antitumor efficacy and circulation kinetics as conventional scFv CAR T cells but offers enhanced targeting control through modulated Fab^LRT^ administration.

### Comparison of GA1CAR T and scFv CAR T cell phenotypes

**Fig. 5.**
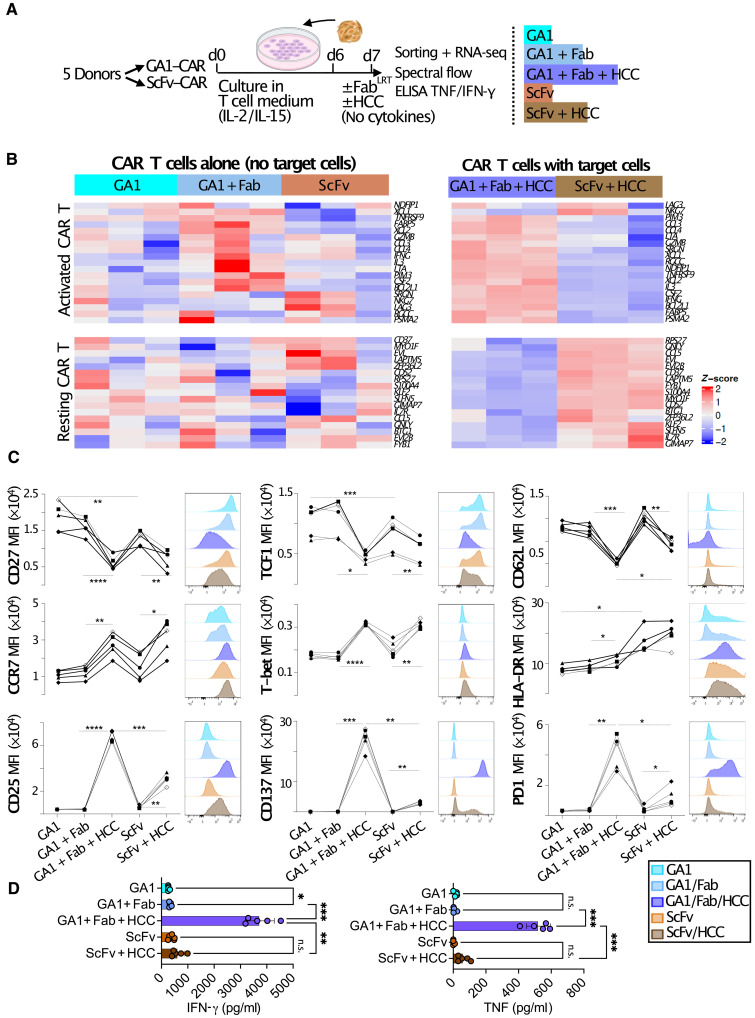
Comparison of GA1CAR T cells and scFv CAR T cell phenotypes. (**A**) Experimental scheme. Donor-matched scFv and GA1CAR CAR T cells were generated from five independent donors. Equal numbers of CAR^+^ cells were activated in vitro with HCC1954 target cells (HCC) with/without anti-Her2 FAB^LRT^ as indicated and analyzed by bulk RNA-seq (after sorting, *n* = 3 donors), spectral flow cytometry, and ELISA. d, day. (**B**) Enrichment in gene signatures indicative of resting or activated status (table S1) (*37*) for scFv and GA1CAR T cell samples. (**C**) Expression of surface and intracellular markers in the different experimental conditions. Each line represents a different donor. Next to each graph, representative histograms illustrate changes in MFI across experimental conditions for that marker in one donor. (**D**) TNF and IFN-γ in supernatant. Each dot represents a different donor. Statistical significance was analyzed using repeated measures ANOVA followed by Šídák’s multiple comparisons tests (*P < 0.05; **P < 0.01; ***P < 0.001); n.s., not significant. The data are presented as the means ± SD; *n* = 5.

In the context of CAR T cell therapy, the expression of different CAR constructs can lead to distinct CAR T cell phenotypes. The scFv CAR is known for low levels of downstream signaling due to tonic signaling, a result of CAR oligomerization caused by interactions between the scFv regions of neighboring CARs (*36*). To explore how GA1CAR expression affects CAR T cell phenotypes compared to scFv CAR, we first conducted bulk RNA sequencing (RNA-seq) on GA1CAR T and scFv CAR T cells isolated from tumors. Matched GA1 or scFv CAR T cells from two independent donors were transferred into groups of five NSG recipient mice each (fig. S11A). On day 6 post-transfer, tumor infiltration by CAR T cells was similar between GA1 and scFv recipients (fig. S11B). RNA-seq analysis was performed on fluorescence-activated cell sorting (FACS)–sorted (fig. S11C) intratumoral T cells from the two donors and two CAR T types, examining gene signatures indicative of resting state versus tonic signaling in in vitro–activated CAR T cells (fig. S11D and table S1) (*37*). Within each donor, ex vivo analyzed GA1CAR T cells seemed to be more enriched in the resting CAR T signature, whereas scFv CAR T cells showed enrichment in the CAR T CD3z tonic signaling signature. However, observations were limited by inter-donor variability. Also, due to the rapid clearance of the Fab in vivo (fig. S9E), the activation status of the GA1CAR T at the time of the analysis (48 hours after the last dose of Fab) was not completely defined. To study phenotypes in more controlled conditions, an in vitro activation model was generated, comparing donor-matched GA1 and scFv CAR T cells from five independent donors (Fig. 5A). These cells were cultured in T cell medium with maintenance cytokines (IL-2/IL-15) for 6 days following CD3/CD28 bead removal. Subsequently, the GA1 and scFv CAR T cells were cocultured with HCC1954 HER2^+^ target cells or maintained in medium (without cytokines) for 1 day. Fab^LRT^ alone was also added to GA1CAR T cells in some wells to assess whether Fab^LRT^ binding by itself could trigger downstream (“tonic”) signaling. FACS-sorted (fig. S12A) CAR^+^ T cells from various conditions underwent bulk RNA-seq (Fig. 5B). Spectral flow cytometry was used to profile the cells using markers associated with resting/memory or activation status at the protein level (Fig. 5C) and effector cytokine production [IFN-γ and tumor necrosis factor (TNF)] was measured in collected supernatants (Fig. 5D).

The gene expression analysis of CAR T cells suggested that adding Fab^LRT^ to GA1CAR T cells, even in the absence of cancer cells, leads to an increased expression of genes related to an activated CAR T status (Fig. 5B, left). In the absence of Fab^LRT^, the differences in gene expression between GA1 and ScFv CAR T cells were small and donor dependent. However, following stimulation with HCC1954 cancer cells (Fig. 5B, right), GA1CAR T cells exhibited an enrichment in expression of the CAR T/T cell activation signature and a decrease in expression of resting CAR T signature compared to scFv CAR T.

We then looked at phenotype differences between GA1 and scFv CAR T cells at the protein level. Unsupervised clustering from spectral flow cytometry surface marker expression data using the X-shift algorithm (*38*) and uniform manifold approximation and projection (UMAP) visualization demonstrated segregation based primarily on the samples’ activation status (fig. S12, B to F). Unstimulated GA1CAR T with/without Fab and unstimulated scFv CAR T had an overlapping distribution in clusters 8, 7, and 1, with the lowest expression of T cell activation markers (e.g., CD25, PD-1, CD137, and TIM-3) and highest expression of markers associated with resting or memory status such as CD62L and CD27 (fig. S12, C to F). In contrast, the distribution of cancer cell–stimulated GA1 CAR T cells (GA1 + Fab + HCC) did not overlap completely with stimulated (ScFv + HCC) CAR T cells, with stimulated ScFv CAR T cells appearing in clusters 1, 4, and 8 at significantly higher rates than GA1 CAR T cells (fig. S12F). ScFv + HCC cells also were present in cluster 3 at lower rates than GA1 + Fab + HCC cells. Because cluster 3 shows the highest expression of certain activation markers (PD-1 and CD137), these data suggest that stimulated scFv CAR T cells display a less activated phenotype than stimulated GA1 CAR T cells. Individual surface (CD27, CD62L, CCR7, HLA-DR, CD25, CD137, and PD-1) and intracellular (TCF1 and T-bet) marker expression was also analyzed across groups after conventional gating (Fig. 5C). Minor differences were observed between unstimulated GA1 and scFv CAR T cells: only a higher TCF1 expression in GA1 (*P* = 0.001) and higher HLA-DR expression in scFv CAR T (*P* = 0.02). The addition of Fab^LRT^ to GA1CAR T cells (GA1 + Fab) did not result in statistically significant protein-level changes in marker expression as compared to GA1 CAR T cells alone. In contrast, exposure to target cells led to substantial changes in protein expression in both types of CAR T cells, particularly an increase in proteins associated with T cell activation and effector function (CD25, CD137, PD-1, and T-bet) and a decrease in molecules linked to resting or memory states (TCF1, CD62L, and CD27). The changes induced by target cell exposure were often more pronounced for GA1CAR T than for scFv CAR T (CD137, PD1, and CD27). Enzyme-linked immunosorbent assay (ELISA) data (Fig. 5D) indicated a significantly higher effector cytokine production for GA1CAR compared to that for scFv CAR T upon target cell stimulation (sixfold increase in IFN-γ, *P* = 0.002; eightfold increase in TNF, *P* = 0.0008). This is likely due to higher levels of surface CAR expression observed in GA1CAR T compared to scFv CAR T (fig. S13, A and B). Collectively, these findings suggest that GA1CAR T cells become more activated upon antigen challenge as compared to scFv CAR T cells.

### Redirecting GA1CAR T cells using Fab^LRT^ with different specificities

GA1CAR T cells can be redirected to multiple targets using Fabs with different antigen specificities. The duration of Fab binding and the effective lifetime of GA1CARs in circulation offer tunable control over their effector activity. This adaptability can be leveraged to alter the target specificity of the CAR T cells in a plug-and-play manner. To test the feasibility of this approach, a model was developed using NSG mice bearing HCC1954 tumors (characterized by low EGFR and high HER2 levels, fig. S13C) treated with GA1CAR T cells alongside anti-EGFR Fab^LRT^ for 10 days (fig. 7). Targeting EGFR slowed tumor growth but did not eradicate HCC1954 tumors due to the low EGFR levels in this cell line. On day 25, half of the mice received a second round of Fab^LRT^ doses targeting HER2. Switching Fabs to target a second antigen allowed GA1CARs to regain control over tumor growth, leading to extended survival for the mice. These results demonstrate the potential of using GA1CARs for targeting multiple antigens following a single infusion of CAR T cells by adjusting the specificity of the Fabs as needed.

## DISCUSSION

This study presents a comprehensive evaluation of the GA1CAR system, a modular CAR designed to enhance the specificity and adaptability of CAR T cell therapies. The GA1CAR design addresses a number of the technical challenges that are faced by traditional CAR T systems, particularly potential toxicity, target heterogeneity, the emergence of antigen-loss variants, and the complexity of manufacturing (*39*). Its design is simpler than most existing universal CARs, with the targeting component, a Fab, being easier to manufacture than a full CAR T product. The results demonstrate the efficacy and versatility of GA1CAR in various in vitro and in vivo settings, highlighting its potential for clinical applications.

The GA1CAR T system is modular and tunable, allowing for the interchange of its targeting moiety in a plug-and-play fashion based on tumor antigen expression profiles. This system offers control over potency and signaling duration to minimize adverse immunological responses. Additionally, it is tag-less, making it less susceptible to cleavage by proteases. Our proof-of-concept studies used a second-generation CAR construct; however, the GA1CAR attributes can be easily integrated into next-generation constructs to leverage ongoing advances in CAR technology.

The GA1CAR system is built on an engineered protein G variant (GA1), which binds effectively to various engineered Fab scaffolds and is non-immunogenic (*27*, *29*). These scaffolds differ by only a few strategically placed mutations, yet produce differences in affinities ranging across three orders of magnitude, allowing for broad manipulation of engagement (*27*, *28*). The engineered Fabs function similarly to single-molecule bispecific T cell engagers, binding tightly and specifically to both the target antigen on cancer cells and the CAR through its GA1 moiety. This dual modulation, by varying Fab affinities or using different affinity scaffolds, offers substantial control over treatment and toxicity management. For example, a lower-affinity scaffold can be used initially and replaced with a higher-affinity scaffold, if well tolerated.

To characterize GA1CAR as a versatile universal system capable of targeting multiple antigens on cancer cells with high selectivity and efficacy, we used immortalized and primary human CD8^+^ T cells alongside breast and ovarian cancer cell lines expressing various endogenous surface antigens. In vivo experiments demonstrated GA1CAR T cells’ effectiveness in two breast cancer models targeting two different antigens (HER2 and EGFR). Sequential administration of Fab^LRT^ with different specificities improved outcomes when initial treatments failed to fully control tumor growth. Further, our results demonstrate that GA1CAR T cells can address tumor escape caused by heterogeneity or low antigen expression by targeting multiple antigens.

Although recent advances in regulatable CAR formats offer high control levels (*22*, *24*, *25*), the conventional scFv CAR format remains the most studied and serves as the standard for comparison. In our mouse xenograft model, GA1CAR showed comparable efficacy in reducing tumors when compared to conventional scFv CARs. While scFv CARs are always loaded due to their covalent attachment to their targeting moiety, leading to potential toxic side effects, the GA1CAR format offers several safety advantages. The Fab^LRT^ used with GA1CAR have a finite lifetime in peripheral blood (~2 days) but can be reloaded as needed through additional dosing. This flexibility contrasts with scFv CARs’ rigidity and associated safety risks from on-target/off-tumor toxicities due to binding similar antigens in healthy tissues. Another key advantage of the GA1CAR system is its ability to target multiple tumor antigens simultaneously or sequentially, reducing the risk of antigen-negative escape. Unlike other systems that use small peptide tags prone to cleavage by proteases, GA1CAR relies on stable protein-protein interactions between GA1 and Fab^LRT^, minimizing susceptibility to cleavage. GA1CAR’s design is also simpler and less structurally constrained than other modular CAR systems.

When comparing transduction rates and expression levels between GA1CAR and scFv CAR constructs generated in the same lentiviral vector, GA1CAR consistently showed higher transduction rates and expression levels in recipient T cells. Adding Fab^LRT^ in the absence of cancer cells partially triggered activation signatures without altering surface marker expression. This suggests that, if tonic signaling occurs, then it has limited consequences at the protein level and depends on the presence of Fab^LRT^. Our universal GA1CAR only recognizes Fabs with LRT or 4D5 mutations, reducing the risk of autoimmune reactions from cross-reactivity with other human antibodies. We anticipate the system’s design will facilitate immunoglobulin G (IgG) formats as targeting molecules. While Fabs offer precise control over dose and activity with better tissue penetration, IgGs provide benefits such as bivalency and longer serum half-life due to FcRn-mediated recycling. Future studies will explore using IgGs with GA1CAR T cells and further optimize hinge, transmembrane, and intracellular domains for specific antigen targets (*40*). Specific spatial orientations between T cells and target cells are crucial for efficient immunological synapse formation, a factor that will be considered in future optimization efforts. Additional strategies could enhance effector functions or safety mechanisms for GA1CAR T cells through co-expression of cytokines or homing receptors or through suicide genes and intracellular split domains. Nonviral GA1CAR delivery methods may also be explored (*41*–*43*).

In summary, we have developed an orthogonal universal CAR T system capable of sequential or simultaneous targeting of multiple cancer antigens while offering enhanced stability, control, and delivery advantages compared to conventional systems. The GA1CAR system can also serve as a preclinical platform for screening Fab^LRT^ molecules from high-throughput antibody discovery pipelines for personalized medicine applications in adoptive cell therapy.

## MATERIALS AND METHODS

### Study design

This study aimed to develop, characterize, and evaluate the functionality of a modular CAR system, termed GA1CAR, designed for enhanced adaptability and tunability in targeting tumor antigens. The modularity was achieved through integration of a specially engineered protein G variant (GA1) with interchangeable antigen-specific Fab scaffolds. To characterize GA1CAR, we assessed binding affinities of various Fab scaffolds to the GA1 module and validated CAR expression and function on lentivirally transduced Jurkat and primary human T cells. Functional assays measured antigen-specific activation using Fabs with varying affinities and multiple target antigens presented on either coated surfaces or cell surfaces. Affinity-dependent effects on T cell responses were explored through IL-2, IFN-γ release, and cytotoxicity assays. To compare GA1CAR and conventional scFv-based CAR T cells, bulk RNA-seq, spectral flow cytometry, and functional cytokine assays were performed to identify differences in tonic signaling, activation profiles, and adaptability in response to antigen engagement.

The in vivo efficacy of GA1CAR T cells was tested in NSG mice bearing HER2^+^ and/or EGFR^+^ human tumors. Tumor regression was monitored following treatment with GA1CAR T cells preloaded with Fabs or receiving sequential Fab administrations to enable antigen-specific reactivation. Pharmacokinetics of GA1CAR T cells and Fab interactions were assessed to inform dosing regimens. Additionally, a plug-and-play approach was tested by redirecting GA1CAR T cells to multiple targets within the same tumor model using Fabs with distinct antigen specificities. Female and Male NSG mice of 8 to 10 weeks of age were randomly allocated to different treatment groups. The investigators were not blinded to group allocation during data collection and analysis. Humane endpoints were used for in vivo experiments in accordance with Institutional Animal Care and Use Committee guidelines. Data are reported for all mice included in each experiment.

### Cell lines

HEK293T, Jurkat, SKBR3, MDA-MB-231, MDA-MB-468, MDA-MB-453, and HCC1954 cell lines were purchased from American Type Culture Collection. HEK-Flp cells were from Invitrogen. The ovarian cancer cells SKOV3ip and OVCAR3 were provided by E. Lengyel at the University of Chicago. Most cells were grown in RPMI 1640 medium with 10% heat-inactivated fetal bovine serum (FBS), penicillin (100 U/ml), and streptomycin (100 mg/ml). HEK293T cells were grown in Dulbecco’s modified Eagle’s medium (DMEM) with 10% heat-inactivated FBS, penicillin (100 U/ml), and streptomycin (100 mg/ml).

### HEK-MBP stable cell

The HEK-MBP stable cell line was created using the HEK Flp-in system following the vendor protocol (Invitrogen, K6010-01). Briefly, a chimeric receptor composed of a mouse IgG kappa signal peptide, full MBP as ectodomain, platelet-derived growth factor receptor transmembrane, and intracellular green fluorescent protein (GFP) was cloned into pcDNA 5/FRT expression vector using Xho I/Xba I cloning sites. The plasmid expressing this chimera (pcDNA 5-MBP/FRT, for simplicity) and the plasmid pOG44 (expressing the Flp recombinase) were electroporated into HEK Flp-in cell line. Stable clones selected with hygromacyn B at 100 μg/ml, and single clones were confirmed by flow cytometry with anti-MBP Fabs and intracellular GFP expression.

### PBMC and CD8^+^ T cell isolation

PBMCs were isolated from leukopak reduction filters obtained by apheresis of de-identified healthy donors from the Blood Bank at the University of Chicago Medicine, via density gradient centrifugation using Ficoll (Corning, 25-072-CV). CD8^+^ T cells were purified using the commercial kit from Miltenyi Biotec, following the instructions suggested. Purity of the separation was monitored by flow cytometry following the manufacturer’s recommendation. CD8^+^ T cells were frozen in 50% FBS, 40% RPMI 1640, and 10% dimethyl sulfoxide (DMSO) at a density of 50 × 10^6^ to 60 × 10^6^ per vial. The final yield of CD8^+^ T ranged from 20 × 10^6^ to 60 × 10^6^ cells per reduction filter

### Lentivirus vector and packaging

The sequence of protein GA1 variant, with a *K33E* mutation to reduce binding to human Fc (*29*) (TPAVTTYKLVINGRTLSGYTTTTAVDAATAE**K**VFKQYAYVHEVDGEWTYDDATKTFTVTEKPEKL), was cloned into a pLVX vector (Takara, 631987) via In-Fusion cloning (Takara, 638944). GA1 sequence was cloned in-frame and upstream of a CD8 hinge, CD28 transmembrane, a 41BB co-stimulatory domain, and CD3ζ domain (full–amino acid sequence in fig. S1). For the scFv control CAR, a HER2-targeting scFv was derived from the commercial Trastuzumab antibody (Protein Data Bank ID: 1N8Z) (*44*). Lentivirus particles were packaged in HEK293T cells plated in four T-225 flasks in HEK medium [10% FBS, DMEM, and penicillin 100 (U/mL)/streptomycin (100 μg/ml)]. The lentivirus vector was co-transfected with psPAX2 (Addgene, 12260) and pMD2.G (Addgene, 12259) using Lipofectamine LTX reagent (Thermo Fisher Scientific, 15338500) at a ratio of 1:0.9:0.21, respectively, with 228 μg of total DNA. Fresh medium was added after 18 hours, and lentivirus particles were harvested at 48 and 72 hours after transfection. Lentivirus was concentrated 10-fold via precipitation with Lenti-X concentrator (Takara, 631232) and resuspended in PBMC medium [RPMI 1640 medium containing 10% FBS, 2× l-glutamine, 1 mM sodium pyruvate, 1× nonessential amino acids (NEAAs) and vitamins, and penicillin (100 U/ml)/streptomycin (100 μg/ml)] before aliquoting and freezing. Viral titer was monitored by Lenti-X GoStix Plus (Takara, 631280) and quantified by quantitative polymerase chain reaction (Takara, 631235).

### Lentivirus transduction

Human CD8^+^ T cells (50 × 10^6^) were plated in a T-175 flask and activated with Dynabeads Human T-Activator CD3/CD28 (Thermo Fisher Scientific, catalog no. 111.31D) for 24 hours, following the manufacturer’s recommendations. After 24 hours of activation, cells were transferred into a six-well plate at 8 × 10^6^ cells per well and transduced with 3.5 ml of concentrated lentivirus particles by spinoculation at 1000*g* for 2 hours at 32°C in medium containing IL-2 at 50 U/μl and polybrene at 6 μg/ml. After transduction, cells were incubated overnight, and fresh medium containing IL-2 (50 U/ml) and IL-15 (50 ng/ml) was added the next morning. Cells were grown for up to 2 weeks before frozen in 50% FBS, 40% RPMI 1640, and 10% DMSO at a density of 50 × 10^6^ to 60 × 10^6^ per vial.

### Cytokine release assay

For cytokine release by GA1CAR transduced in Jurkat cells, ELISA plates (Greiner Bio-One, 655081) were coated for 2 hours with neutravidin (2 μg/ml) and 1 hour with MBP-biotin (100 nM). Next, 10 × 10^5^ GA1CAR cells per well were incubated with Fab at 10 nM overnight. Additionally, 10 × 10^5^ GA1CAR cells were incubated with 1 × 10^5^ HEK-MBP target cells and different Fab scaffolds and concentrations. Cells were cocultured overnight in a 96-well U-bottom shape plate (Greiner Bio-One, 650261) in fresh medium without cytokines. The next day, supernatants were collected and analyzed for human IFN-γ and IL-2 using commercial kits from Cisbio (catalog nos. 62HIFNGPEH and 62HIL02PEH, respectively). In the case of human CD8^+^ T cells, 10 × 10^5^ CD8T cells transduced with GA1CAR and 1 × 10^5^ target cells were cocultured overnight in a 96-well U-bottom shape plate in fresh medium without cytokines. The next day, supernatants were collected and analyzed for human IFN-γ and IL-2 using commercial kits from Cisbio.

### Cytotoxicity assay

Cytotoxicity was assessed by measuring the release of lactate dehydrogenase (LDH) into the culture medium. Briefly, effector and target cells were cocultured at effector-to-target ratios of 30:1, 10:1, 3:1, 1:1, and 0.3:1 in triplicate in U-bottom 96-well plates. Each well contained 20 × 10^3^ target cells in a final volume of 200 μl. The cells were cultured in RPMI 1640 medium supplemented with 10% FBS. Cells were incubated with various effector cells and Fab fragments at a final concentration of 1 nM for 16 hours at 37°C in 5% CO_2_. Following incubation, supernatants were collected for LDH quantification using the CytoTox 96 Non-Radioactive Cytotoxicity Assay (Promega, catalog no. G1780), according to the manufacturer’s instructions. For cell lysis control, 20 μl of lysis buffer was added to designated wells 45 min before harvest.

### scFv/GA1CAR T cell activation in vitro

CD8^+^ T cells were isolated from five healthy donors and subsequently used to generate donor-matched GA1 and scFv CAR T cells. Following CD3/CD28 bead activation and transduction, CAR T cells were expanded in RPMI 1640 medium supplemented with 10% FBS, 1% penicillin/streptomycin (P/S), 2 mM l-glutamine, 1 mM sodium pyruvate, NEAAs (1×), IL-2 (50 U/ml), and IL-15 (50 ng/ml) for 10 days post-transduction. On day 10, human T cell activator CD3/CD28 beads were removed, and cells were washed twice with cytokine-free medium. GA1CAR T cells were then incubated with or without HER2 Fab^LRT^ for 30 min in cytokine-free medium. GA1CAR T cells incubated or not with HER2 Fab^LRT^ (200 nM) and HER2-specific scFv CAR T cells were cocultured with HCC1954 cells in duplicates, in 12-well tissue culture plates in a volume of 1.5 ml per well for 24 hours at 37°C in a 5% CO_2_ incubator. The CAR T to target cells ratio was 3:1 (5 × 10^6^ CAR T cells to 1.6 × 10^6^ target cells). After 24 hours, cell supernatants were collected and stored at −80°C for subsequent cytokine analysis using BioLegend ELISA Kits: TNF (catalog no. 430204) and IFN-γ (catalog no. 430104). Half of the cells from each sample were allocated for single-cell RNA-seq after extraction of RNA using the RNeasy Mini Kit (QIAGEN), and the remaining half was used for immunophenotyping using flow cytometry.

### Intratumoral scFv/GA1CAR T cell analysis ex vivo

Donor-matched GA1 and scFv CAR T cells were injected intravenously into HCC1954-tumor bearing NSG mice at day 12 after tumor inoculation, at 4 million CAR^+^ cells per mouse. Two different donors were used and five recipient mice per group, for a total of 4 groups and 20 mice. GA1-recipient mice were treated with three doses of HER2 Fab^LRT^ intraperitoneal (200 μg per dose) at days 0, 2, and 4 since GA1CAR T cell transfer. At day 6, all mice were euthanized. Tumors were excised, weighed, cut into 1-mm fragments, and digested for 25 min at 37C with Liberase (75 μg/ml; Roche) and deoxyribonuclease I (20 μg/ml; Sigma-Aldrich) under gentle shaking. Cell suspensions were filtered using 70-μm cell strainers. Half of each individual mouse sample was used for quantification of infiltrating CD8^+^ T cells per gram of tumor by flow cytometry by staining with Live/Dead Yellow Dead Cell Stain (Invitrogen) and antihuman CD8 APC (BioLegend) and acquisition using a Cytek Aurora flow cytometer. The other half of each individual tumor sample was pooled per group (i.e., cells from the five mice from each group were pooled into a single sample) before magnetic enrichment for human CD8^+^ T cells using CD8 MicroBeads from Miltenyi Biotec (130-045-201). Pooled CD8-enriched samples were then stained with Live/Dead Yellow Dead Cell Stain and antihuman CD8 APC. CD8^+^Live/Dead^−^ cells were sorted in a FACSAria. Pellets were processed, and RNA was extracted according to the instructions of the RNeasy Micro Kit (QIAGEN).

### Flow cytometry

Cell staining in Fig. 1 was done using different Fab concentrations in cell medium (10% FBS, RPMI 1640, and P/S) at 37°C for 30 min, followed by two washes, and secondary antibody Alexa Fluor 647 staining (Jackson ImmunoResearch, catalog no. 109-605-006) at 400× dilution and at 4°C. CD69 expression in Fig. 2 was measured by flow cytometry using an anti-CD69 antibody conjugated to APC (BioLegend, 310910). Briefly, 0.1 × 10^6^ to 0.2 × 10^6^ cells were stained in 50 μl of 1:40 antibody dilution in 1× PBS buffer, incubated for 30 min, washed twice with 1× PBS, and fixed in 0.5% paraformaldehyde before flow cytometry analysis.

**Fig. 6.**
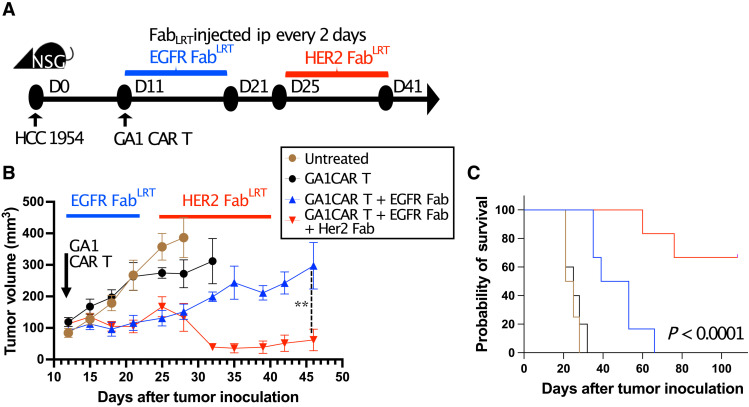
Switching Fabs to redirect GA1CAR T cells to different targets in vivo. (**A**) Experimental scheme. HCC1954 tumor-bearing mice received GA1CAR T cells with or without treatment with EGFR Fab^LRT^ for 10 days. After that, half of the EGFR Fab^LRT^-treated animals received a second round of Fab^LRT^ for 16 days, this time specific for HER2, whereas the other half received no further treatment. *n* = 5 for control groups (untreated, GA1CAR only), *n* = 6 for Fab-receiving groups (EGFR only and EGFR + HER2). D, day; ip, intraperitoneally. (**B**) Tumor volumes per group (average ± SEM). Mann-Whitney test was performed at day 46 comparing EGFR and EGFR/HER2 groups. **P < 0.01. (**C**) Kaplan-Meier survival plot was analyzed with log-rank (Mantel-Cox) test.

For GA1 and scFv phenotyping (Fig. 6), cells were washed with PBS before staining with Live/Dead Blue Fixable dye (Invitrogen, no. L34961A) for 30 min at room temperature, washed with PBS, stained with surface antibodies for 15 to 30 min at 4°C, washed again, and fixed using Cytofix (BD Biosciences, catalog no. 554655). GA1CAR T samples were preincubated with an irrelevant Fab^LRT^ at 200 nM for 30 min before staining with an anti-F(ab′)_2_ antibody for detection of the GA1 CAR. For intracellular staining, the Foxp3/Transcription Factor Staining Buffer Set (catalog no. 00-5521-00) from Invitrogen was used according to the manufacturer’s instructions. The following antihuman antibodies were used for CAR T cell immunophenotyping: anti-CCR7 BV421 (catalog no. 353208), anti-CD62L BV605 (catalog no. 304833), anti-CD127 BV650 (catalog no. 351325), anti-CD25 BV711 (catalog no. 302635), anti-Fas BV785 (catalog no. 305645), anti-CD27 fluorescein isothiocyanate (FITC; catalog no. 302806), anti-CD52 PerCP-Cy5.5 (318911), anti-TIM-3 PE (catalog no. 345006), anti-CD137 PE-Dazzle 594 (catalog no. 309825), anti–HLA-DR PE-CY7 (catalog no. 307607), anti-PD-1 PE-CY7 (catalog no. 329917), anti-CD39 APC/Fire 750 (catalog no. 328229), anti-Ki67 BV711 (catalog no. 350515), and anti-T-bet FITC (catalog no. 644811) from BioLegend; anti-TCF1/TCF7 PE (catalog no. 14456S) and anti-LEF1 Alexa Fluor 700 (catalog no. 38921S) from Cell Signaling Technology; anti-F(ab′)_2_ Alexa Fluor 647 (catalog no. 109-606-097) from Jackson ImmunoResearch laboratories; and anti-CD8 BUV805 (catalog no. 612889) from BD Biosciences. NSG mouse blood samples were surface stained, treated with NH4Cl red blood cell lysis buffer, and immediately acquired (without washing) after addition of CountBright beads (Invitrogen) to determine the absolute counts of CAR T cells in peripheral blood. Samples were analyzed using a Cytek Aurora spectral flow cytometer (ex vivo/in vitro phenotyping) or BD Fortessa (circulating CAR T analysis) at the University of Chicago Cytometry and Antibody Technology Core. Flow cytometry data were analyzed using FlowJo v10.10 software (FlowJo LLC, BD Life Sciences, Ashland, OR, USA). For unsupervised clustering analysis of flow cytometry data, 10^5^ CAR^+^ cells per sample were concatenated and used for downstream analysis. FlowJo plugin X-shift (*38*) was used for unsupervised nearest-neighbor clustering based on phenotypic similarities. High-dimensional data were visualized using UMAP plugin v4.0.4, and Cluster Explorer plugin was used for cluster visualization and marker expression mapping on UMAP plots.

### Single-molecule pulldown assay, spot counting, and photobleaching analysis

Previously described methods were followed (*30*, *45*). In brief, quartz slides were passivated with methoxy polyethylene glycol (PEG; 200 mg/ml) containing 2.5% (w/w) biotinylated PEG. HEK293 cells were transfected with CAR constructs (1.5 × 10^6^ cells in six-well plate) and lysed after 48 hours in 200 μl of lysis buffer [50 mM tris (pH 7.4), 120 mM NaCl, 1% NP-40, 5 mM EDTA, 25 mM NaF, 25 mM sodium pyrophosphate, 25 mM β-glycerophosphate, 1 mM phenylmethylsulfonyl fluoride (PMSF), leupeptin (1 μg/ml), chymostatin (1 μg/ml), pepstatin (1 μg/ml), and antipain (1 μg/ml)]. Cell lysate was cleared by centrifugation at 10,000*g* for 10 min and diluted 100-fold or enough to obtain a surface density optimal for single-molecule analysis. For SNAP tag labeling, 100 μl of non-diluted lysate was labeled with 1 μl of 0.6 mM dye, incubated for 30 min at 37°C, and pass through a spin column (PD MiniTrap G-10, Cytiva) to remove free dye, and fractions were collected and used for single-molecule pulldown. Mean spot count per image and SD were calculated from images taken from 10 or more different regions. For photobleaching analysis, 500 frame videos were recorded using a 200-ms exposure, and single-molecule fluorescent time traces of surface immobilized proteins were manually scored as having one to four bleaching steps or were discarded if no clean bleaching steps could be identified. Total number of molecules successfully scored as bleaching in one to four steps (*n*) is depicted in the figures. Single-molecule data were collected in a custom-built total internal reflection fluorescence microscope equipped with a 100× 1.40 numerical aperture objective (Olympus) and EMCCD camera (iXon, Andor Technology). All experiments were performed at room temperature (22° to 25°C).

### Analysis of gene expression

RNA samples were analyzed by The University of Chicago Functional Genomics Facility (RRID:SCR_019196). RNA quality and quantity was assessed using the Agilent bio-analyzer. Strand-specific RNA-seq libraries were prepared using a TruSeq mRNA-seq library preparation protocol (Illumina provided). Library quality and quantity was assessed using the Agilent bio-analyzer, and libraries were sequenced using an Illumina NovaSEQ6000 (Illumina provided reagents and protocols). RNA-seq data were preprocessed using the nf-core (*46*) rnaseq v3.14.0 pipeline (https://doi.org/10.5281/zenodo.1400710). Briefly, read quality control was performed using FastQC v8.30, read alignment using STAR v2.7.9a, duplicate marking using Picard v3.0.0, and read quantification using Salmon v1.10.1. The pipeline was executed with the following command: nextflow run nf-core/rnaseq -r 3.14.0 -profile singularity --input sample_info.csv --max_memory 48.GB --genome GRCh38G34 --gencode --skip_preseq false --skip_rseqc true --pseudo_aligner salmon --outdir results -resume. Raw reads were then converted to counts per million, filtered for low gene expression, and normalized by library size using trimmed mean of *M*-value normalization in R v4.2.1 using the EdgeR v.3.38.4 package.

### Fab cloning

Fab against MBP has been previously reported by our lab (*32*). Fab against HER2 was cloned as described previously (*28*). Fabs against PRLR were generated by phage display selection using recombinant prolactin receptor fused to Fc fragment. Briefly, the humanized CDR-containing regions were cloned into pSFV4 or pRH2.2 vectors, via In-Fusion cloning. Fabs against EGFR and MSLN were cloned using the heavy chain variable (VH) and light chain variable (VL) chains sequences published in #US 6235883B1 and #WO2015090230A1 patents, respectively. All VH and VL sequences (including LRT mutations) were fused upstream of CH and CL chains and were chemically synthesized, as gBlocks by Integrated DNA Technologies. The gBlocks were inserted into pRH2.2 vector via In-Fusion cloning for protein expression.

### Fab purification

Fabs were cloned in pRH2.2 or pSFV4 vectors [isopropyl-β-d-thiogalactopyranoside (IPTG) inducible] for bacterial expression. Fabs were expressed in the periplasm of *Escherichia coli* BL21 (Gold). Cells were grown in 2× YT medium for 4 to 5 hours at 37°C. After induction by 1 mM IPTG at 0.8 to 1.2 optical density at 600 nm, cells were harvested by centrifugation and sonicated in 50 mM tris (pH 7.5), 200 mM NaCl, and 2 mM PMSF. The centrifugation cleared lysates were heated at 60°C for 30 min. Next, the lysate was centrifuged 40 min at 4000*g* to remove precipitated proteins. Lysates were filtered through a 0.2-μm filter and purified on AKTA purifier equipped with a homemade Protein GA1 resin (SulfoLink Coupling Resin, Thermo Fisher Scientific). Next, peak fractions were loaded onto an ion exchange Resource S 1-ml column (GE Healthcare). After washing with 50 mM sodium acetate (pH 5.0), Fabs were eluted with a linear 0 to 100% gradient of buffer containing 50 mM sodium acetate (pH 5.0) and 2 M sodium chloride. Fractions containing pure Fab were pooled, neutralized with 50 mM Hepes (pH 7.5), dialyzed against buffer containing 50 mM Hepes (pH 7.5) and 200 mM sodium chloride or 1× PBS, concentrated, and stored in aliquots at −80°C.

### Endotoxin removal and quantification

Endotoxin removal from Fabs was done with Proteus NoEndo columns (GEN-NoE24Micro; Protein Ark), and final levels quantified using the Limulus Amebocyte Lysate assay (88282; Thermo Fisher Scientific). The final endotoxin levels in the Fab preps were below 5 endotoxin units/mg.

### Xenograft mouse model

Animal care and use were in accordance with the institutional and National Institutes of Health protocols and guidelines. All studies were approved by the Animal Care and Use Committee of The University of Chicago. All mice were maintained under specific pathogen–free conditions. Six- to 8-week-old female and male NSG (NOD. Cg-Prkdc^scid^ Il2rg^tm1Wjl^/SzJ) mice were purchased from the Jackson Laboratory and/or bred in house. HCC1954 or MDA-MB-231 cells (3 × 10^6^ to 5 × 10^6^) in growth factor–reduced Matrigel at 1:1 v/v (BD Biosciences, catalog no. 356231) were injected subcutaneously into the right flank of mice. Once tumor reached a volume of 100 to 150 mm^3^, mice received 10 × 10^6^ to 20 × 10^6^ of CAR + scFv or GA1CAR T cells by retro-orbital injection preincubated or not with α-HER2 Fab^LRT^ for 30 min, followed by a dose of anti-HER2 Fab^LRT^ every other day for 10 to 14 days. In some experiments, mice received anti-EGFR Fab^LRT^_._ All Fab^LRT^s (in 1× PBS buffer) were injected intraperitoneally at 200 μg per mouse. In control groups, mice were injected with GA1CAR T cells only or received no treatment. Tumors were measured with a caliper and tumor volume calculated as WxHxL/2. In HCC194-bearing mice, tumors in untreated control groups often experience spontaneous ulceration and/or leakage of fluid when they reach 300- to 500-mm^3^ volume and need to be euthanized; therefore, we used a 300-mm^3^ tumor volume endpoint for the survival plot in Fig. 6C. Peripheral blood was obtained from the retro-orbital sinus under general anesthesia or via tail vein venipuncture. Blood (25 to 30 μl) was used for each flow cytometry analysis on circulating CAR T cells.

### Statistical analysis

All experimental data are presented as means ± SD of at least three sets of independent experiments, unless otherwise indicated. Data analysis was performed using GraphPad Prism software (GraphPad Software, San Diego, CA). Number of independent repeats and statistical test/s used for analysis are specified on each figure legend.
