# Supplementary material for: A universal chimeric antigen receptor (CAR)–fragment antibody binder (FAB) split system for cancer immunotherapy
Source: Sci Adv. 2025 Jul 4;11(27):eadv4937. doi: 10.1126/sciadv.adv4937 (PMC12227065; doi:10.1126/sciadv.adv4937)
Supplement: Supplementary file 1 — Figs. S1 to S13 Table S1 [file sciadv.adv4937_sm.pdf]

Science Advances
AAAS

Supplementary Materials for

A universal chimeric antigen receptor (CAR)–fragment antibody binder (FAB) split system for cancer immunotherapy

Ainhoa Arina *et al.*

Corresponding author: Anthony A. Kossiakoff, koss@bsd.uchicago.edu; Ainhoa Arina, aarina@bsd.uchicago.edu

Sci. Adv. **11**, eadv4937 (2025)
DOI: 10.1126/sciadv.adv4937

This PDF file includes:

Figs. S1 to S13
Table S1

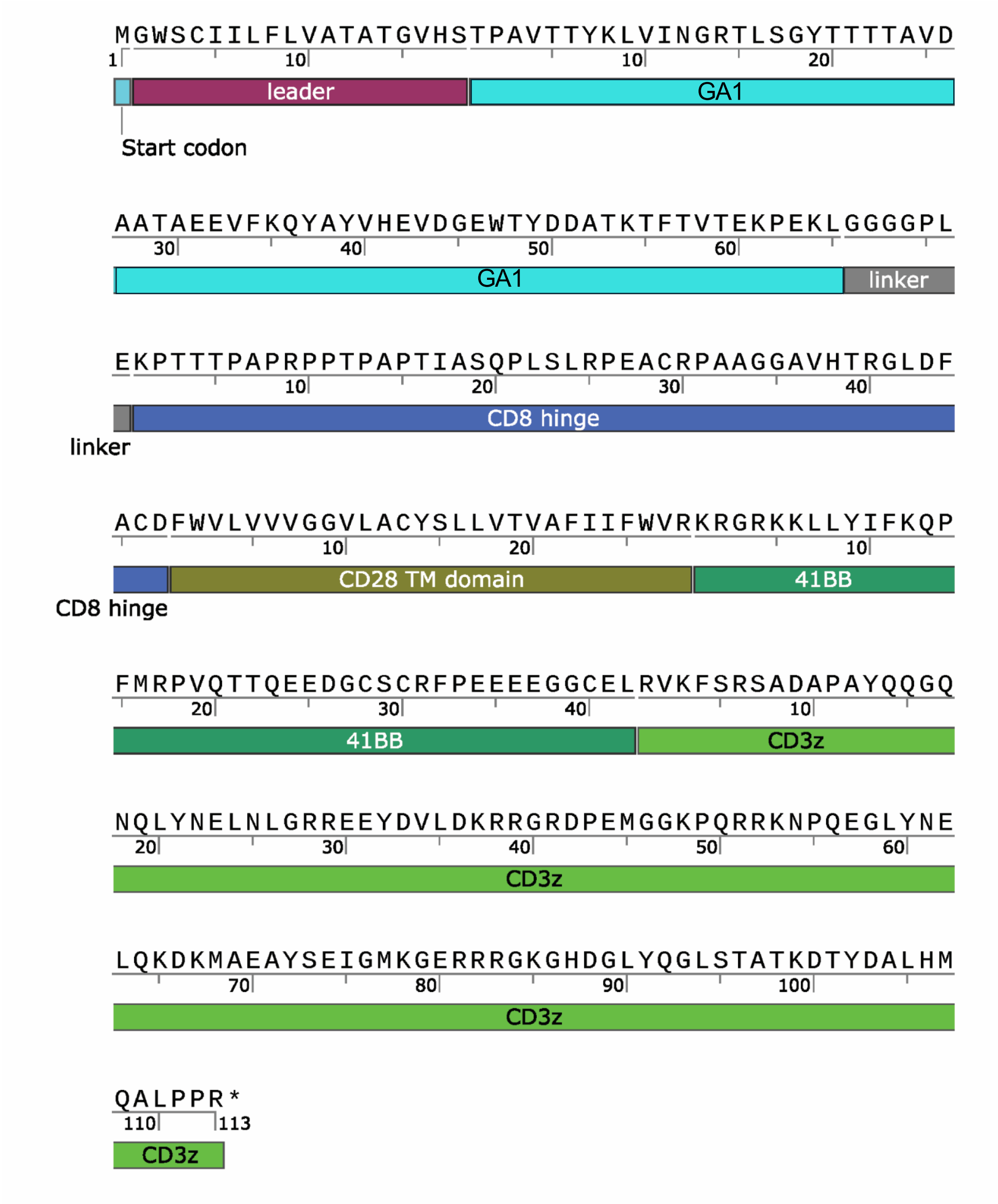

Fig. S1. GA1CAR amino acid map sequence.

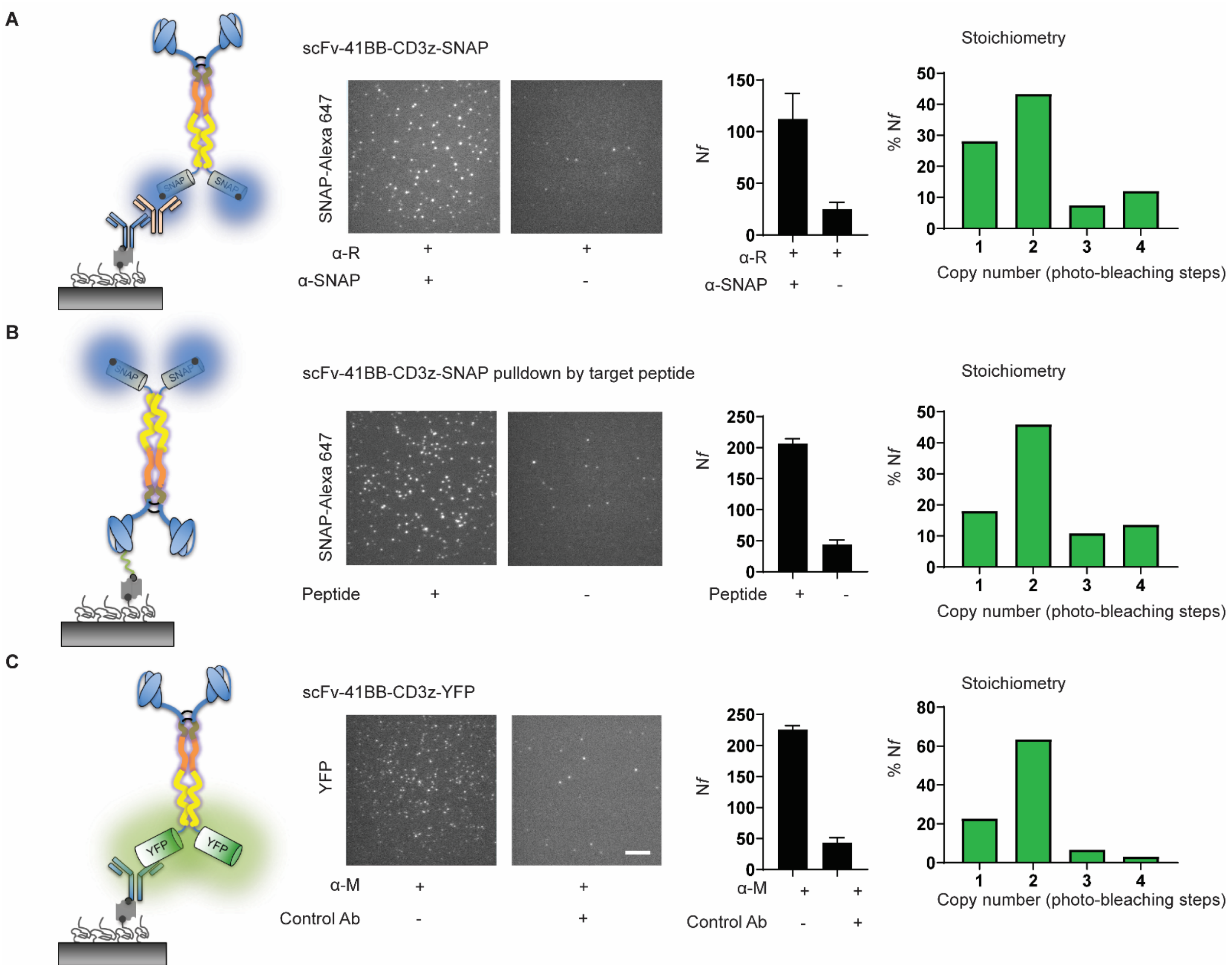

Fig. S2. GA1CAR is dimeric. Schematic depiction of pulldown, representative fluorescent images, average number of molecules per imaging area (N*f*), and distribution of fluorescent photo-bleaching steps (stoichiometry) from **(A)** CAR-T pulldown via anti SNAP rabbit antibody and anti-R antibody; **(B)** CAR-T pulldown via biotinylated peptide that bind to scFv in CAR-T; **(C)** CAR-T pulldown via anti-YFP mouse biotinylated antibody. Scale bar 5 µm.

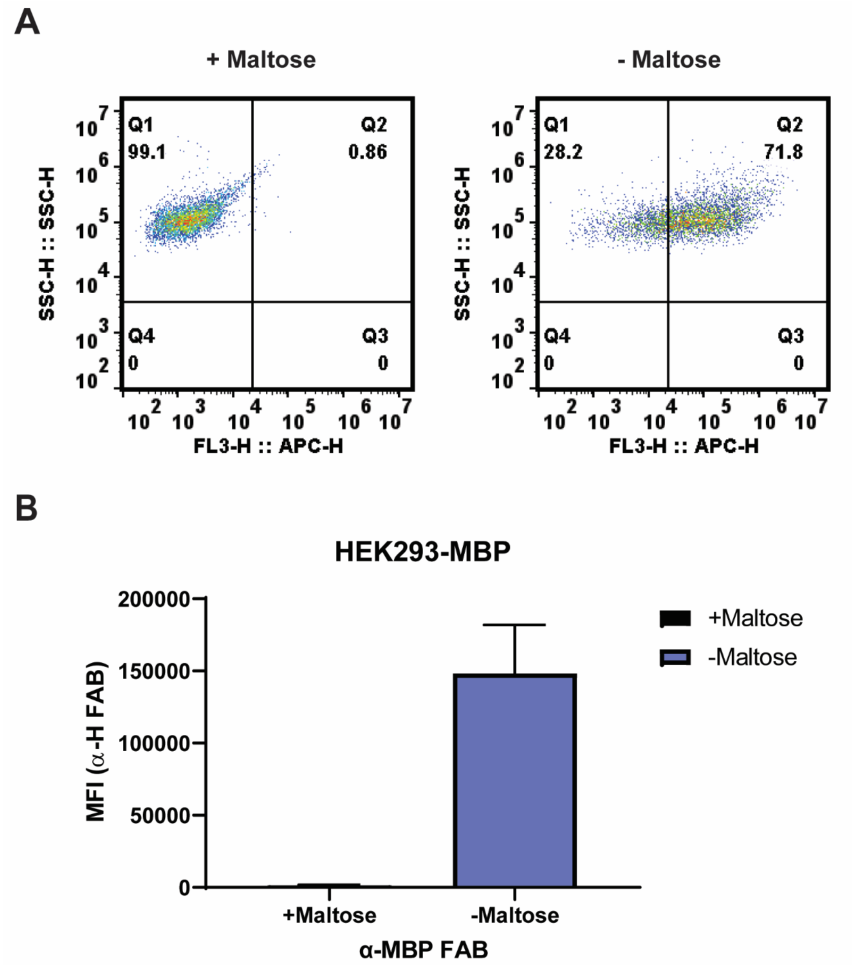

Fig. S3. Expression of MBP on HEK-Flp cells. **(A)** Representative flow plots showing expression of MBP on the surface of HEK 293 cells, detected using an anti-MBP Fab^{LRT} and a secondary anti-human Fab antibody conjugated to Alexa Fluor® 647. Binding of conformation-specific anti-MBP Fab^{LRT} to MBP decreases in the presence of maltose. **(B)** Quantification of binding as determined by the mean fluorescent intensity (MFI). Anti-MBP Fab^{LRT} was used at 50 nM. The data are presented as the mean ± SD, n = 3.

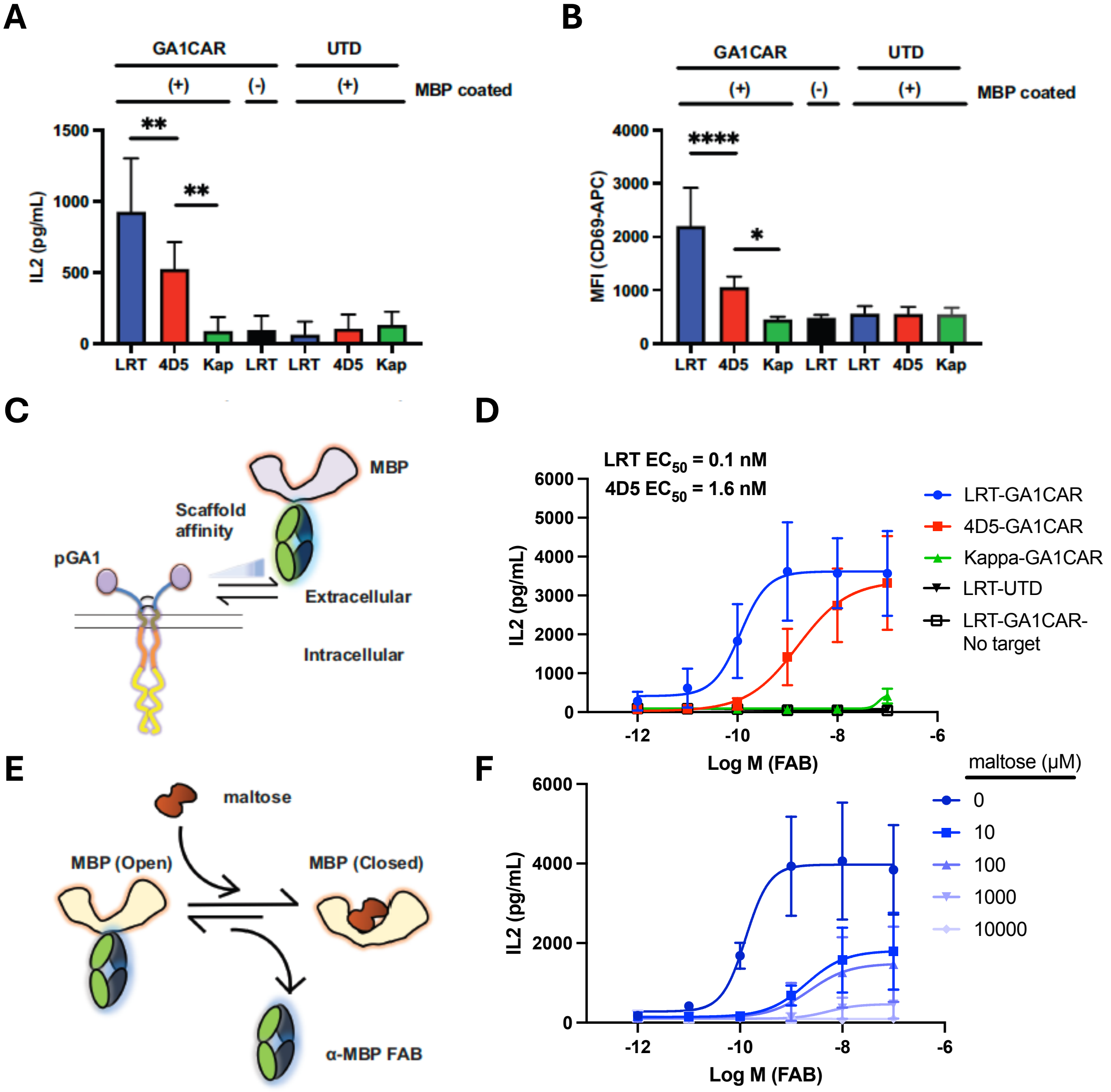

Fig. S4. Functional characterization of GA1CAR expressed in Jurkat T cell lymphoma cells. (A) IL2 production **(B)** CD69 expression by GA1CAR-Jurkat cells after culture in MBP-coated plates with different anti-MBP Fab scaffolds **(C)** Cartoon shows Fab scaffolds with different affinities for GA1CAR. **(D)** IL2 release by GA1CAR-Jurkat cells after 16h co-culture with HEK-MBP cells at increasing concentrations of anti-MBP Fab scaffolds. **(E)** Cartoon showing the conformation-specific anti-MBP Fab binding to MBP. The affinity decreases with increasing maltose concentration **(F)** Affinity dependent release of IL2 by GA1CAR-Jurkat cells in the presence of HEK-MBP cells and varying concentrations of maltose. Statistical significance was determined by Tukey's test after one-way ANOVA (*$P < 0.05$; **$P < 0.01$; ****$P < 0.0001$). Data represents the mean ± SD, n = 3.

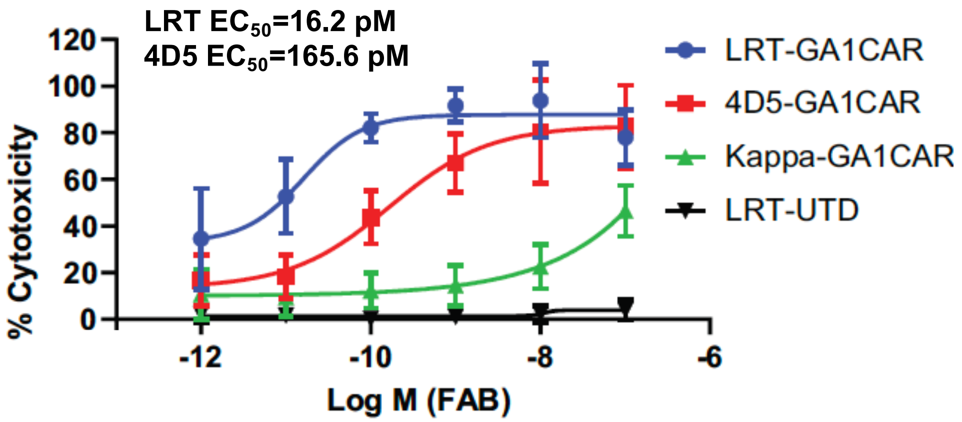

Fig. S5. GA1CAR-T cell cytotoxicity when incubated with different concentration of anti-MBP Fab scaffolds and HEK-MBP cells. The data are presented as the mean ± SD, n = 3.

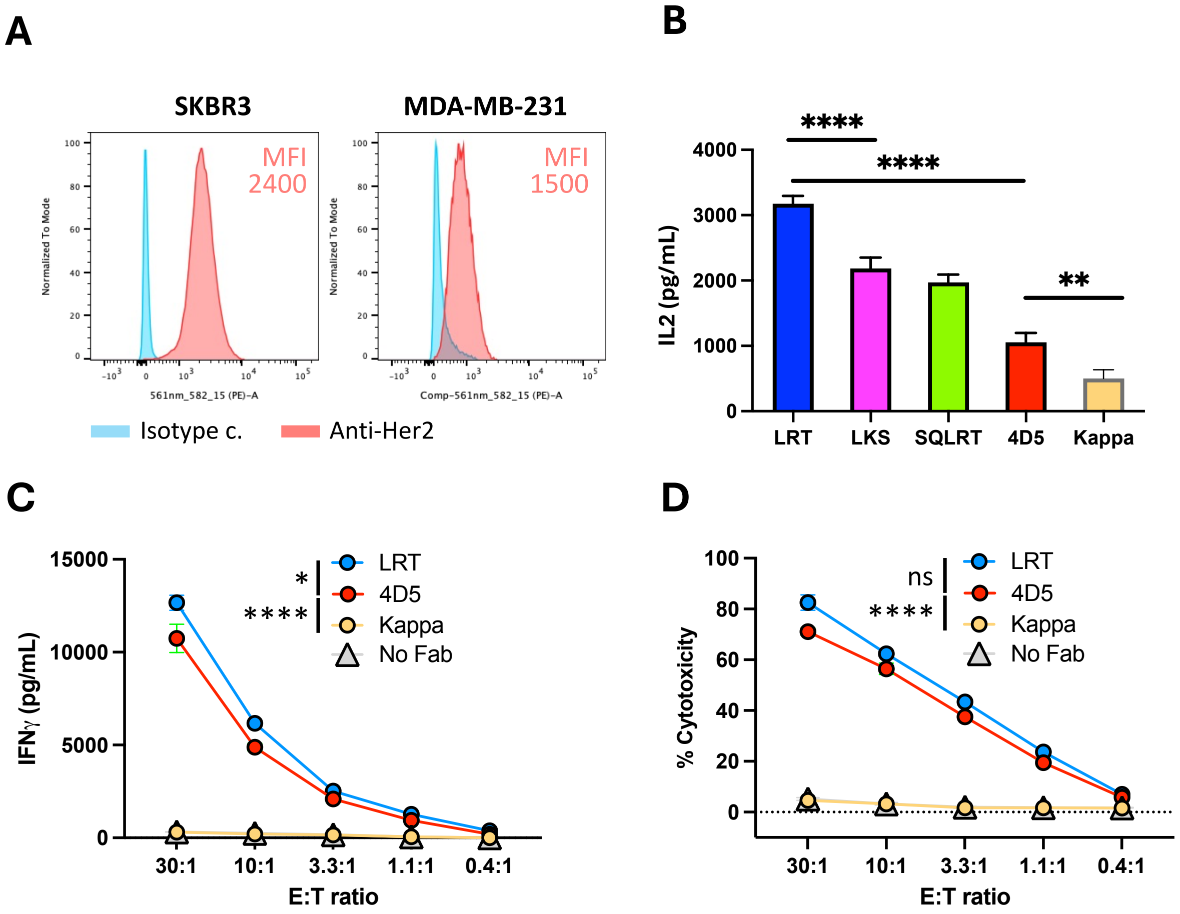

Fig. S6. GA1CAR-T cell function using anti-HER2 Fab scaffolds with different affinities for GA1CAR and MDA-MB-231 cells as targets. (A) HER2 expression is lower in MDA-MB-231 as compared to SKBR3 cells. **(B)** IL2 release (E:T ratio=10:1), **(C)** IFNγ release, and **(D)** Cellular cytotoxicity when incubating GA1CAR-T cells with MDA-MB-231 cells and anti-HER2 Fab scaffolds of decreasing affinities (LRT>LKS>SQLRT>4D5). Kappa scaffold is used as negative control. Statistical significance was determined by Tukey's multiple comparisons test after one-way ANOVA **(B)** or two-way ANOVA test **(C, D)**. Statistical significance shown in C,D for the most relevant comparisons is at the 10:1 ratio. (*$P < 0.05$; **$P < 0.01$; ****$P < 0.0001$). The data are presented as the mean ± SD, n = 3.

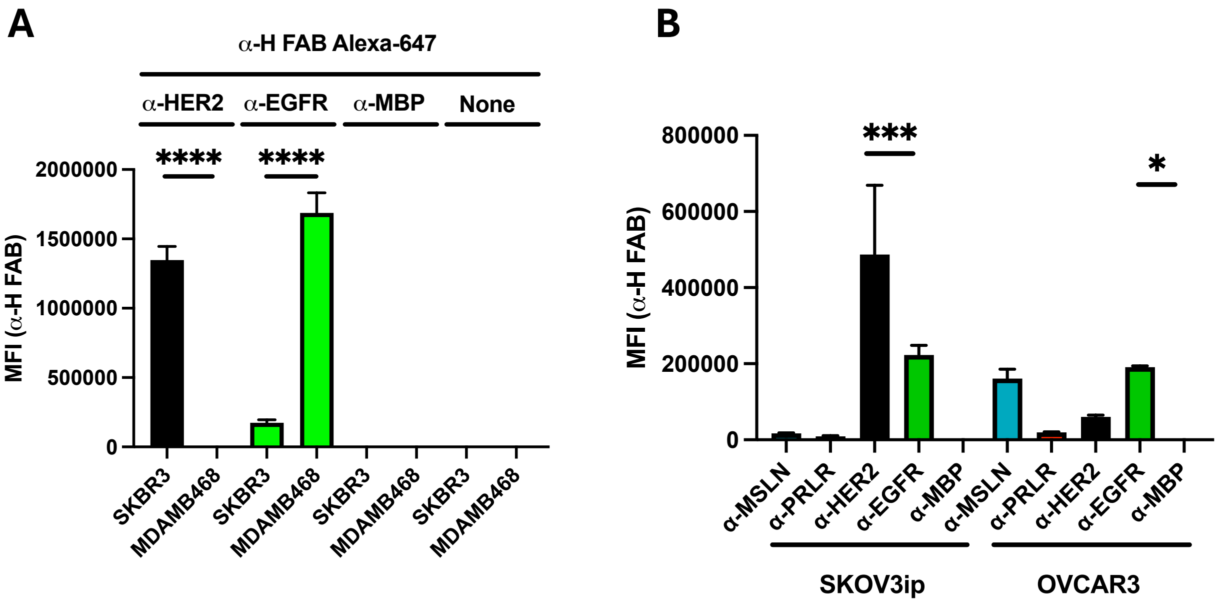

Fig.S7. Quantification of cancer antigen expression on different target cells. (A) Expression of HER2 and EGFR in SKBR3 and MDAMB468 breast cancer cell lines, determined by flow cytometry. **(B)** Quantification of several ovarian cancer antigens on SKOV3ip and OVCAR3 cells by flow cytometry. Antigen expression was detected using antigen-specific Fab^{LRT} and a secondary anti-human Fab antibody conjugated to Alexa Fluor® 647. Statistical significance was determined by Tukey's multiple comparisons test after one-way ANOVA (*$P < 0.05$; ***$P < 0.001$; ****$P < 0.0001$). The data are presented as the mean ± SD, n = 3.

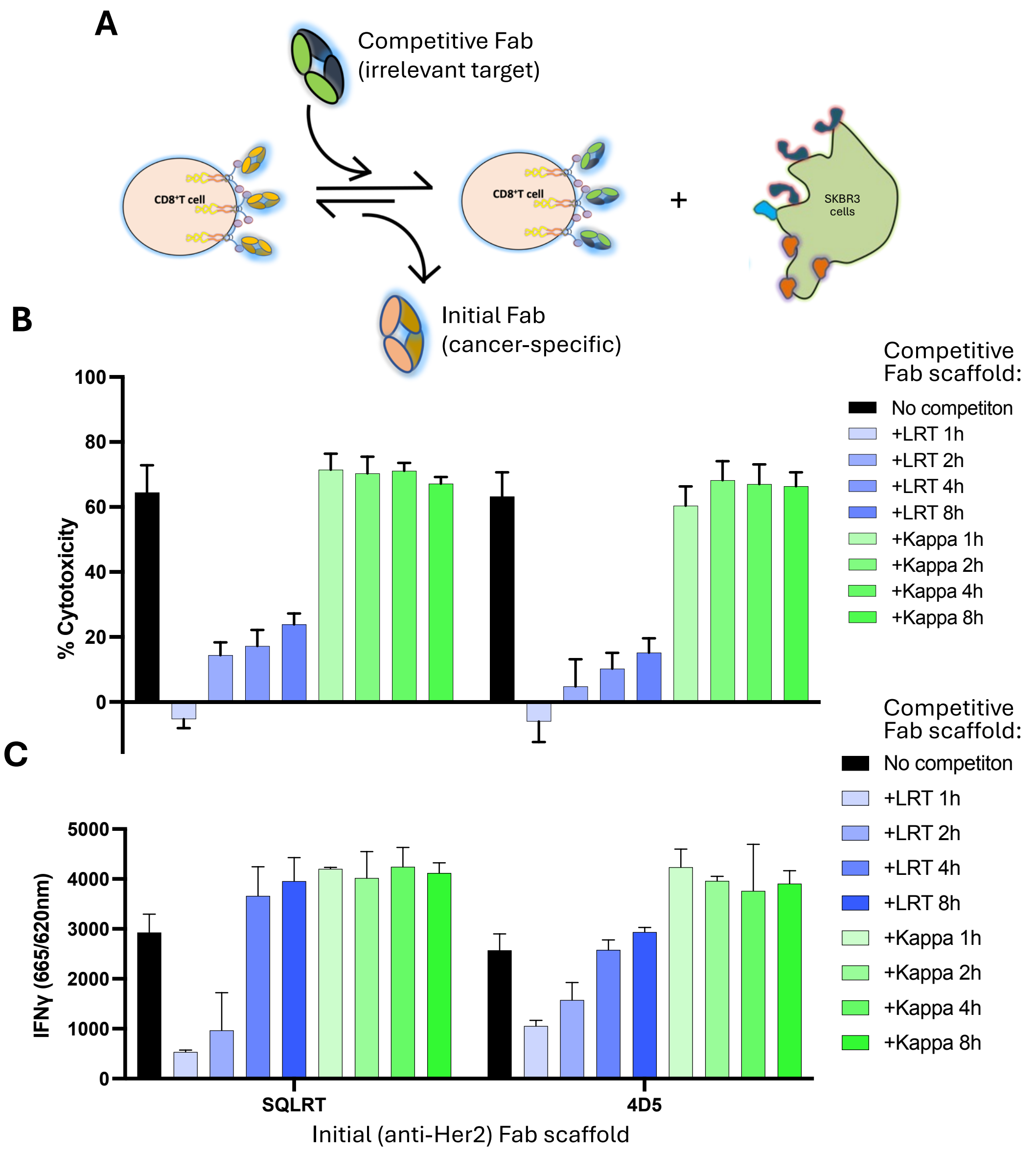

Fig. S8. GA1CAR-T emergency stop switch. (A) Model of the GA1CAR-T emergency stop switch. A competitive Fab scaffold with a higher affinity to GA1 can compete and deactivate the GA1CAR-T cells. HER2$^+$ SKBR3 cancer cells were attached to the plate overnight, followed by addition of the GA1CAR-T cells and 100 nM of HER2 Fab in either SQLRT or 4D5 scaffolds the next day. Cell killing by LDH assay **(B)** and IFNγ release **(C)** were measured after 24h. To test the effect of the emergency stop switch, 1 μM of the competitive isotype Fab in the LRT scaffold was added after 1h, 2h, 4h or 8h (isotype Fab recognizes SARS-CoV-2 RBD protein). The same isotype Fab in the Kappa scaffold was added as a negative control. We observed a reduction of cell killing upon CAR-T detachment from the HER2 Fabs in both SQLRT and 4D5 scaffolds (blue bars) compared to no emergency switch off (black bar) or control Kappa scaffold (green bar). The data are presented as the mean ± SD, n = 3.

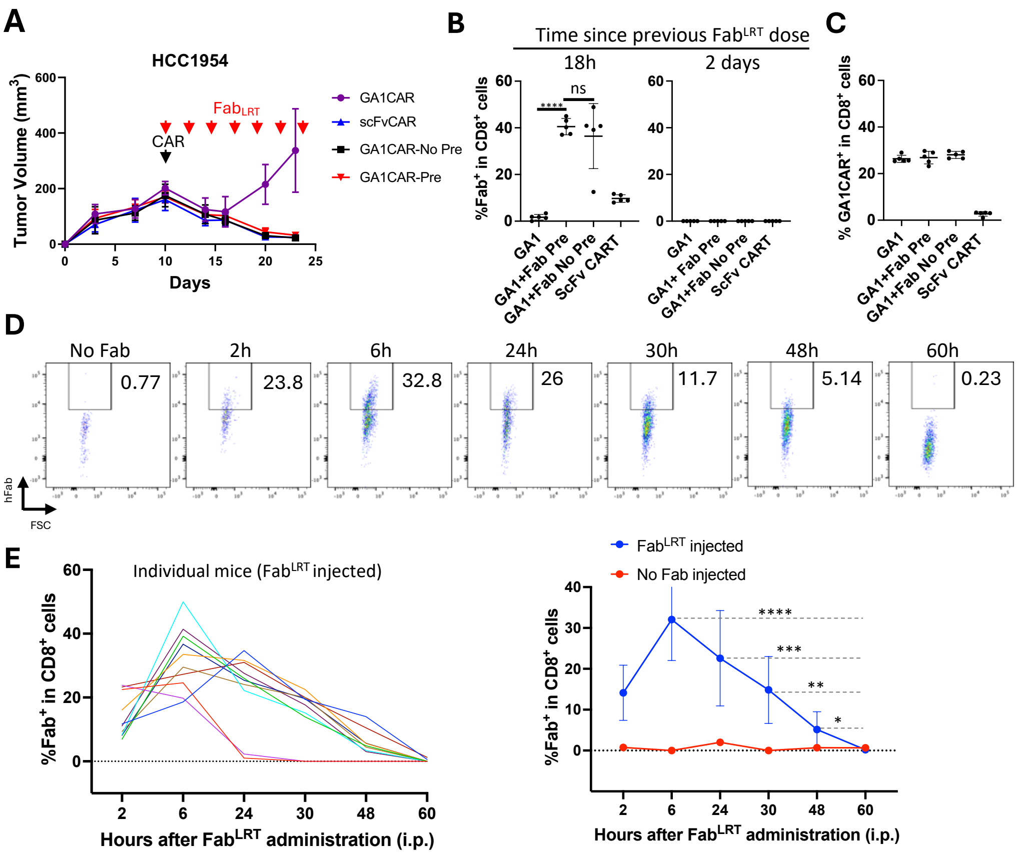

Fig. S9. Pre-incubation with Fab^{LRT} is not required for effectiveness of GA1CAR-T cells in vivo and kinetics of Fab^{LRT} bound to circulating GA1CAR-T cells. (A) HCC1954 tumor-bearing mice were injected i.v. at day 10 with GA1CAR-T cells that had been preincubated with HER2 Fab^{LRT} for 30 min before injection (Pre) or not preincubated (No Pre). The latter group of mice received Fab^{LRT} i.p at day 10. Both groups received 6 additional i.p. doses of Fab^{LRT} given every other day. Control groups included mice treated with GA1CAR-T cells but no Fab^{LRT}, and mice treated with scFv CAR-T cells (N=5/group). **(B)** PBL were directly stained with an anti-human F(ab')$_2$ antibody to detect Fab^{LRT} bound to GA1 in vivo, 18 h after receiving the first dose of Fab^{LRT} and two days after the third dose. **(C)** PBL were preincubated with an irrelevant Fab^{LRT} (anti-MBP) and then stained with an anti-human F(ab')$_2$ antibody to measure GA1 expression. **(D-E)** Kinetics of in vivo binding of Fab^{LRT} to GA1CAR. Ten HCC1954 tumor-bearing mice were injected with 10x10^6 GA1CAR-T cells i.v and a single dose of 200 ug Her2 Fab^{LRT} i.p. PBL were directly stained with an anti-human F(ab')$_2$ antibody at the indicated times. **(D)** Representative plots showing %Fab$^+$ cells in gated human CD8$^+$ cells at each time point. **(E)** GA1CAR/Fab binding in the individual mice over time (left panel) or as mean and SD of the 10 animals (right panel). Two control mice received GA1CAR-T cells but no Fab^{LRT} . Statistical significance was determined by one-way ANOVA (*$P < 0.05$; **$P < 0.01$; ***$P < 0.001$; ****$P < 0.0001$), ns: not significant.

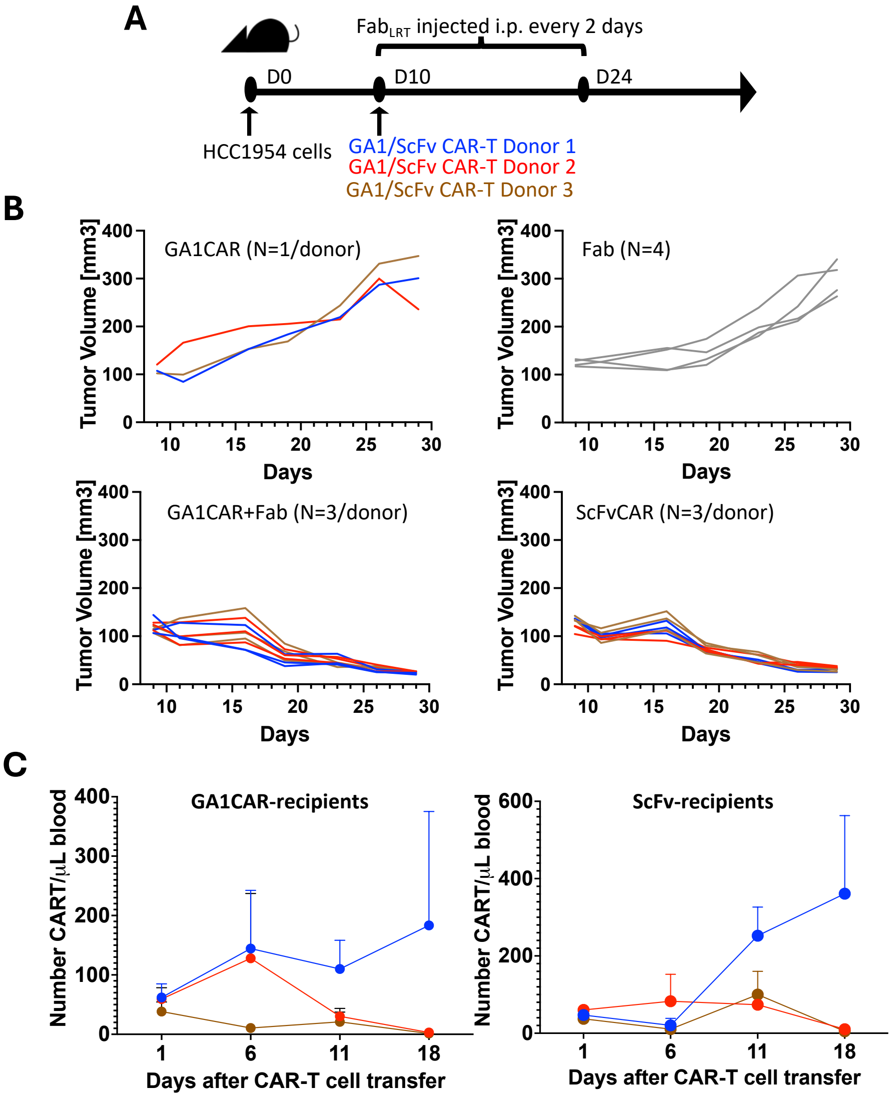

Fig. S10. Kinetics of circulating CAR-T cells and of tumor elimination are similar for mice treated with GA1 and scFv CAR-T cells. (A) Experimental scheme. HCC1954-bearing NSG mouse cohorts were treated with donor-matched GA1+Fab^{LRT} or scFv CAR-T cells, from three independent human donors. Control groups received only GA1CAR-T cells or only Fab^{LRT}. **(B)** Tumor growth. Each line is an individual mouse, and colors (blue, red, brown) indicate different donors. **(C)** Absolute numbers of circulating CAR-T cells were determined at different time points after CAR-T cell transfer, for each CAR-T cell type and donor. The data are presented as the mean ± SD, n = 3 recipient mice per T cell donor and time point.

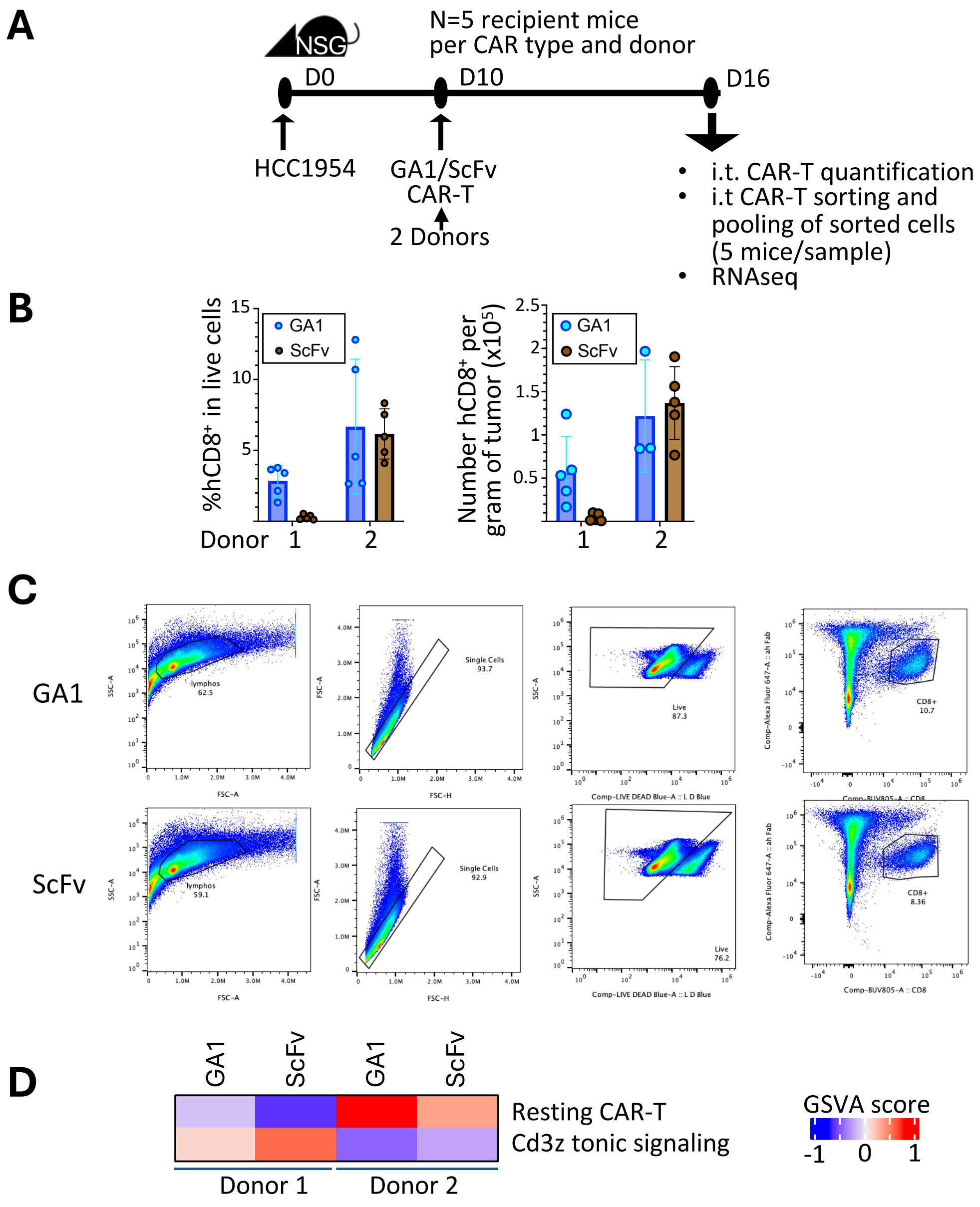

Fig. S11. Ex vivo analysis of intratumoral GA1CAR-T cells vs. scFv CAR-T cells. (A) Experimental scheme. Groups of 5 NSG mice were treated with donor-matched scFv/GA1CAR-T cells from two independent donors (4 groups total). **(B)** Tumor-infiltrating CAR-T cells were quantified by flow cytometry at day 6 after CAR-T cell transfer. **(C)** Gating strategy for sorting and RNAseq of ex vivo isolated intratumoral CAR-T cells. **(D)** Enrichment in gene signatures indicative of resting/tonic signaling status from Boroughs et al. 2020 (37) was compared between FACS-sorted tumor-infiltrating scFv and GA1CAR-T cells by bulk RNAseq analysis. Gene lists for each signature are in Table S1.

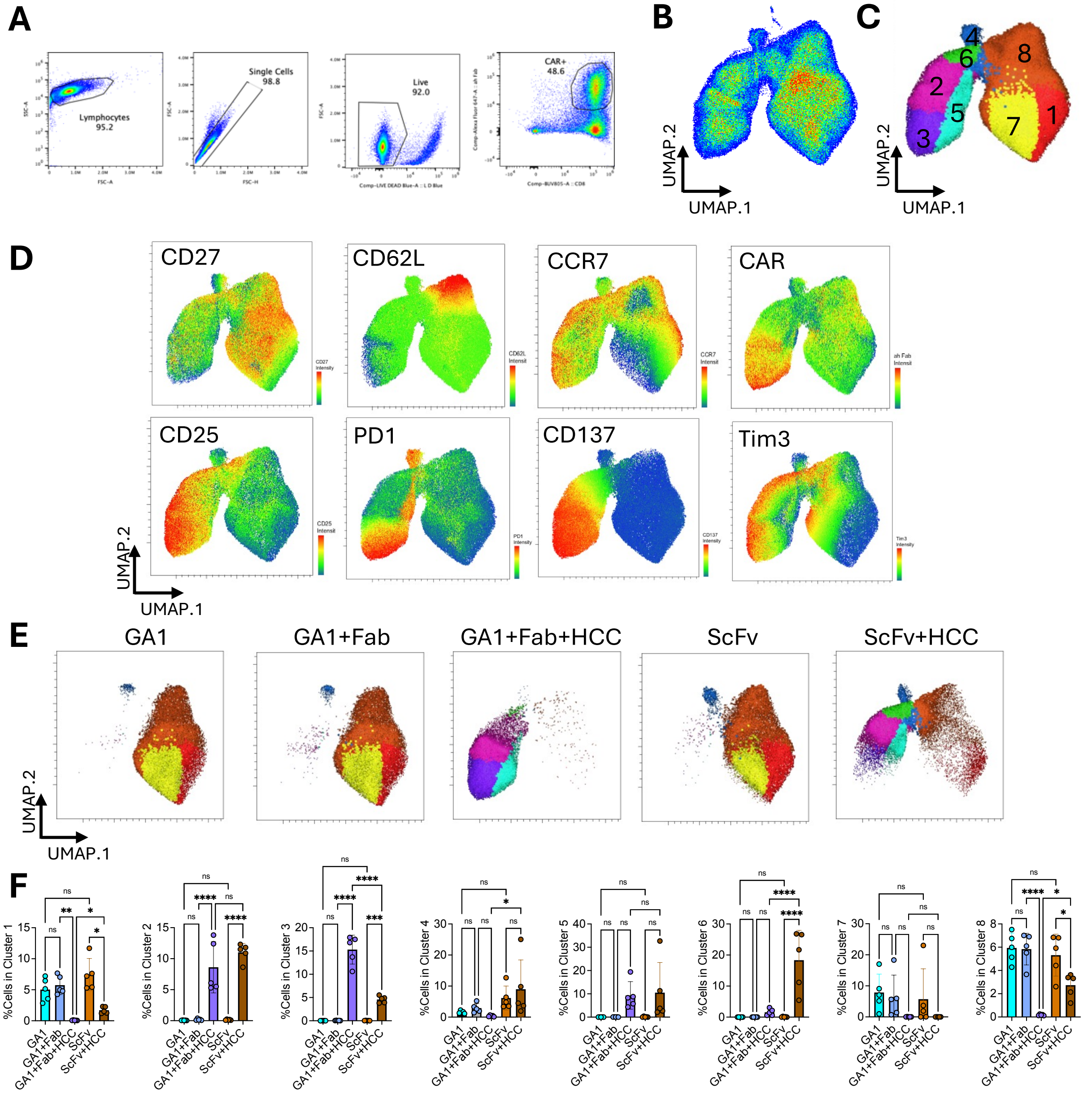

Fig. S12. Gating strategy for sorting and unsupervised clustering analysis of in vitro cultured GA1 and scFv CAR-T cells. (A) Gating strategy for sorting of GA1 and scFv CAR-T cells cultured in vitro and RNAseq from Fig. 5B. **(B-F)** Unsupervised uniform manifold approximation and projection (UMAP) clustering of 250,000 live CAR^{+} cells (10,000 cells per sample, 5 donors, 5 experimental conditions) **(B)** UMAP visualization **(C)** Unsupervised clustering using X-shift algorithm resulted in 8 clusters based on differential expression of markers shown in D. **(D)** Intensity of individual marker expression in UMAP plots. **(E)** UMAP plots of cells gated by experimental condition. Each plot corresponds to pooled samples from 5 donors for the indicated group (50,000 cells per group). **(F)** Distribution of cells from the 25 total samples into clusters. Each dot corresponds to an individual donor.

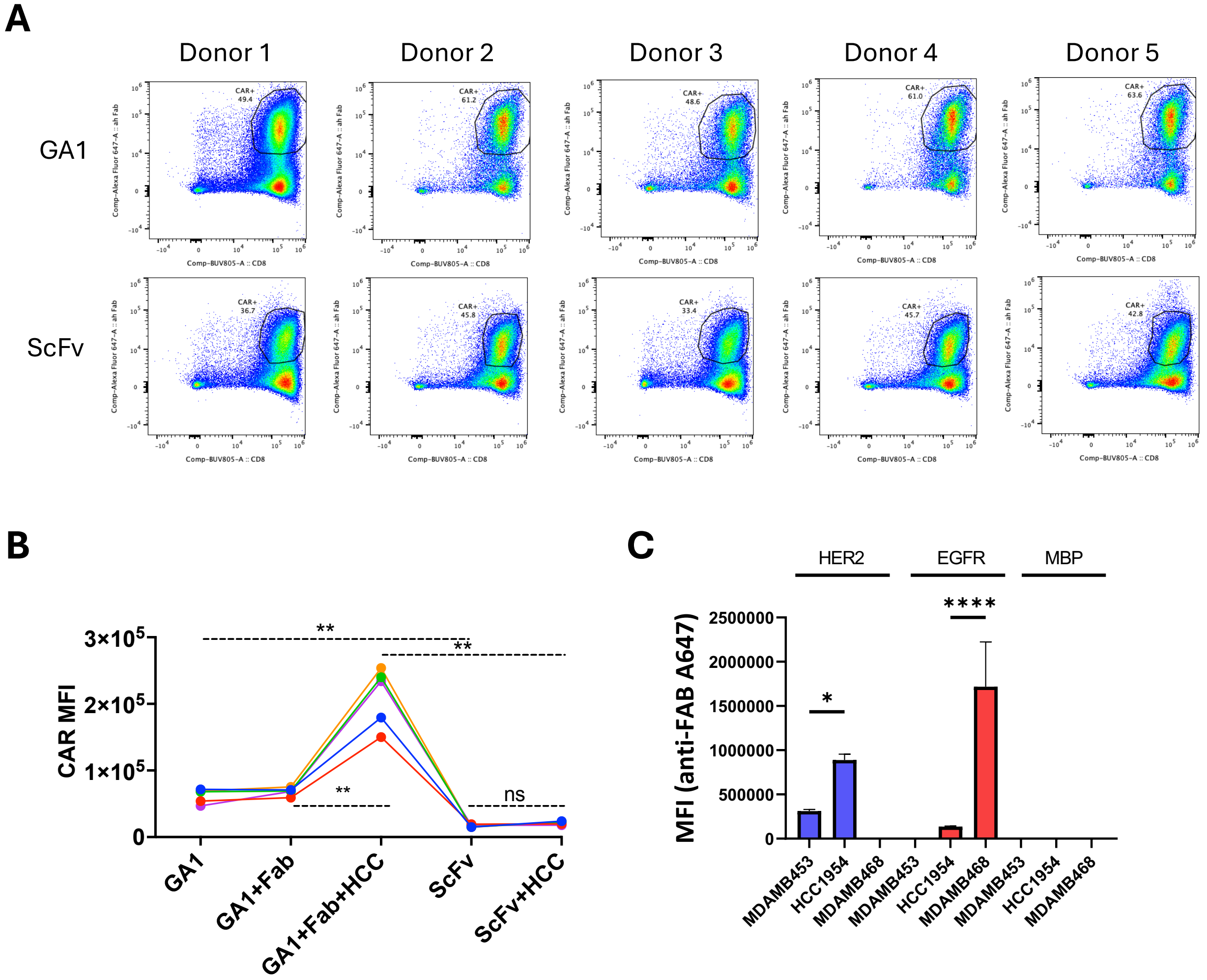

Fig. S13. Variations in CAR expresion on CAR-T cell surface according to CAR type (A) and experimental condition (B) and relative expression of HER2 and EGFR on breast cancer cell lines (C). (A) GA1CAR and ScFv CAR-T cell cultures were generated from five individual donors. Donor 3 GA1 CAR-T cell gating is also shown in Fig. S12 as a representative example. In **(B)**, each colored line represents a different donor. **(C)** HER2 and EGFR expression was determined by flow cytometry on HCC1954 and MDA-MB-453 cells using antigen-specific Fab^{LRT} followed by a secondary anti-human $F(ab')_2$ antibody conjugated to Alexa Fluor 647. MDA-MB-468 cells were used in parallel as a negative control for HER2 expression and positive control for EGFR expression. Statistical significance on (B) was analyzed using repeated measurements ANOVA followed by Šídák's multiple comparison tests and on (C) by Tukey's multiple comparisons test after one-way ANOVA (**$P < 0.01$, *$P < 0.05$; ****$P < 0.0001$, ns:not significant). Data are presented as mean ± SD, n = 5 (B) and n = 3 (C).

Table S1. Gene signatures used for RNAseq analysis

Signature	**Resting CAR-T**	**Cd3ζ Tonic signaling**	**CAR-T cell activation**
Source	**Ref. (37), Table S1, "No Stim"**	**Ref. (37), Fig. 2A, "Up"**	**Ref. (37), Table S2, column A, "Up in Nalm6 Stim vs Unstimulated""**
Gene list	CCL5	ASB2	IFNG
	CD52	BIRC3	IL3
	IL7R	CCL3	CCL4
	BTG1	CCL4	XCL1
	GIMAP7	GGT1	CSF2
	S100A4	CTLA4	XCL2
	FYB (FYB1)	CSF2RB	CCL3
	EVL	GZMB	LTA
	SLFN5	ZP3	GZMB
	KLF2	SDC4	LAG3
	RPS27	XCL1	TNFRSF9
	MALAT1	ZBED2	PIM3
	MYO1F	IFNG	RGCC
	CD37		NKG7
	LAPTM5		FABP5
	FAM65B		NDFIP1
	ZFP36L2		MIR155HG
	RARRES3		SRGN
	EVI2B		PSMA2
	GNLY		BCL2L1
